# Anti-Herpes Simplex Virus (Wild-Type and Drug-Resistant) Properties of Herbal Kerra^TM^, KS^TM^, and Minoza^TM^

**DOI:** 10.3390/v17070889

**Published:** 2025-06-24

**Authors:** Chaleampol Loymunkong, Kiattawee Choowongkomon, Chukkris Heawchaiyaphum, Nutchanat Chatchawankanpanich, Chamsai Pientong, Tipaya Ekalaksananan, Jureeporn Chuerduangphui

**Affiliations:** 1 Department of Microbiology, Faculty of Science, Kasetsart University, Bangkok 10900, Thailand; chaleampol.lo@ku.th; 2 Department of Biochemistry, Faculty of Science, Kasetsart University, Bangkok 10900, Thailand; kiattawee.c@ku.th; 3 Department of Microbiology, Faculty of Medicine, Khon Kaen University, Khon Kaen 40002, Thailand; chukhe@kku.ac.th (C.H.); chapie@kku.ac.th (C.P.); tipeka@kku.ac.th (T.E.); 4 HPV & EBV and Carcinogenesis Research Group, Khon Kaen University, Khon Kaen 40002, Thailand; 5 Medical Life Sciences Institute, Department of Medical Sciences, Ministry of Public Health, Nonthaburi 11000, Thailand; nutchanat.c@dmsc.mail.go.th

**Keywords:** herbal medicines, acyclovir, herpes simplex virus

## Abstract

Commercial herbal compounds are a main attractive target to explore for a novel drug for the treatment of HSV. This study investigated the anti-HSV infectivity of extracts derived from the Thai commercial herbals Kerra^TM^, KS^TM^, and Minoza^TM^. Wild-type HSV-1 KOS, HSV-2, and drug-resistant HSV-1 dxpIII were used to investigate any inhibitory effects of these extracts. A plaque formation assay was performed to investigate the effects of all extracts. The viral *ICP4*, *UL30*, *gD*, and gB and cellular *IL1β*, *IL6*, *STAT3*, and *NFKB1* expression levels were evaluated. The Kerra^TM^, KS^TM^, and Minoza^TM^ extracts at 50–200 μg/mL significantly inhibited HSV-1 KOS and dxpIII infection in the post-entry step, whereas only Minoza^TM^ could not reduce plaque formation of HSV-2. In addition, *ICP4*, *UL30*, and *gD* mRNAs and gB protein were significantly decreased in Kerra^TM^- and KS^TM^-treated cells. Furthermore, *IL1B*, *IL6*, *STAT3*, and *NFKB1* expression was upregulated in Kerra^TM^- and KS^TM^-treated cells. Kerra^TM^ and KS^TM^ could be agents against HSV infection, especially the HSV acyclovir (ACV)-resistant strain. From the docking result and drug-likeness prediction, 2-Methoxy-9H-xanthen-9-one, piperine, and sargassopenilline D found in Kerra^TM^, KS^TM^, and Minoza^TM^ show high binding energy closely resembling ACV, and are desirable as drug-like characteristics.

## 1. Introduction

Infection with herpes simplex virus (HSV) can cause mild (cold sores, genital herpes, and ulcers) to highly severe symptoms (encephalitis) and is associated with Alzheimer’s disease, and is found in all age ranges, especially immunocompromised individuals [1]. This virus is transmitted by close contact including sexual transmission and the intrapartum period. Primarily, HSV infects and is replicated in the epithelium, subsequently infecting peripheral neurons and migrating to nerve ganglia, causing viral latent infection [2,3]. HSV latent infection can cause the recurrence of cold sores throughout life because there is no elimination of HSV-infected nerve ganglia. In particular, immunocompromised and immunocompetent patients are more frequently infected with HSV and need to be treated with acyclovir (ACV), an anti-HSV drug, with such treatment possibly leading to drug resistance by HSV [3].

The replication cycle of HSV is initiated by viral glycoprotein gD binding to the host receptor, leading to the activation of gH and gB, which triggers membrane fusion and subsequent viral internalization. The viral capsid and tegument proteins, including VP16, are released into the cytoplasm, and traverse to the cell nucleus along microtubules contributing to viral DNA and protein released into the nucleus [4,5]. The mRNA expression (1–4 h) of viral immediate early (IE) genes (e.g., *ICP0* and *ICP4*) is induced by VP16, and then IE proteins (2–4 h) are translated to promote mRNA and protein expression of early (E) genes (2–7 h), including *UL30* encoding DNA polymerase, to replicate the viral genome, which plays an important role in viral replication, and is a target of several drugs against HSV, including ACV as well as other natural compounds [6]. Then, E proteins induce late (L) gene expression (>3 h) encoding viral structural proteins (e.g., gD, gB, and gC). Viral genomes are encapsidated after L proteins and HSV DNA are sufficiently produced. Finally, the mature virus leaves the nucleus and cell membrane (6–18 h) [4,5].

HSV is divided into types 1 and 2, depending on serology, genetics, and the anatomical site of clinical manifestation [7]. Primarily, HSV-1 infects the oral region via oral-to-oral contact and is associated with orolabial herpes or a cold sore, whereas HSV-2 almost always infects the genital tract and causes genital herpes. However, HSV-1 can infect and cause disease via oral–genital transmission [8]. Furthermore, HSV-2 is rarely found in the oral region via genital-to-oral contact [9]. HSV has infected more than 3 billion people globally with HSV-1 and HSV-2 [8].

Latent infection with HSV is a major problem in immunocompromised patients and can require long-term treatment that may contribute to viral mutation. ACV is a gold standard drug for herpes labialis; therefore, cidofovir and foscarnet as alternative drugs are necessary for treatment in ACV-resistant strains [10]. However, these drugs have a high risk of adverse effects [11].

There are many reports of natural products that can be used instead of or synergistically with ACV in clinical trials, including *Allium hirtifolium*, *Andrographis paniculata*, *Clinacanthus nutans*, olive leaf, licorice, sumac, and lemon balm extracts [12,13,14,15,16]. In addition, the biological activity of many extracts from natural products inhibits HSV via various pathways, for example, the regulation of cellular cytokines, including interferon beta (IFNβ), tumor necrosis factor alpha (TNFα), interleukin-1 (IL-1), and IL-6, with viral and host transcriptional factors: ICP0, ICP4, and nuclear factor kappa B (NF-κB) [17,18,19,20]. Notably, treatment with some natural products increased antiviral cytokines IFNβ, TNFα, IL-1, and IL-6, which are negatively correlated with decreasing HSV-1 reproduction [17,18].

The current study focused on the use of commercial Thai herbal extracts Kerra^TM^, KS^TM^, and Minoza^TM^ on anti-HSV wild-type and drug-resistant strains to explore novel potential anti-HSV agents.

## 2. Materials and Methods

### 2.1. Cell Culture

An African green monkey kidney cell line (Vero) kindly provided by Prof. Dr. Pilaipan Puthavathana (Mahidol University, Thailand) was cultured in a complete medium including Dulbecco’s Modified Eagle Medium/high glucose (Gibco Laboratories, Grand Island, NY, USA), 10% fetal bovine serum (FBS) (Gibco Laboratories, Grand Island, NY, USA), 40 μg/mL gentamicin, 2.5 μg/mL amphotericin B, 100 μg/mL streptomycin, and 100 unit/mL penicillin G and incubated in 5% CO_2_ in an incubator at 37 °C.

### 2.2. The Extraction of Kerra^TM^, KS^TM^, and Minoza^TM^

Samples of powder (100 g) of each of Kerra^TM^, KS^TM^, and Minoza^TM^ (Vetchakorn Osot, Bangkok, Thailand) provided according to the previous study were added along with 200 mL of 99.5% ethanol and shaken at 150 rpm overnight [21]. After passing through Whatman no. 1 filter paper, the extract was centrifuged at 12,000 rpm and 4 °C for 10 min. Then, the supernatant was collected and evaporated using a rotary evaporator and then lyophilized. The dried pellet was dissolved in dimethyl sulfoxide (DMSO) and stored at −20 °C.

### 2.3. Viruses

The HSV-1 strain KOS and HSV-2 clinically isolated strains were kindly provided by Prof. Dr. Pilaipan Puthavathana (Mahidol University, Thailand). Prof. Donald Coen (Biological Chemistry & Molecular Pharmacology, Harvard Medical School, Boston, MA, USA) kindly provided HSV-1 dxpIII (a phosphonoacetic acid- and phosphonoformate-resistant strain). All viral strains were propagated and titered in Vero cells and stored at −80 °C.

### 2.4. Cytotoxicity

Vero cells were seeded in a 96-well plate at 10^4^ cells/well and maintained for 24 h. Each extract was added to the cells at various concentrations and incubated for 48 h. Ten microliters of 5 mg/mL 3-(4,5-dimethylthiazol-2-yl)-2,5-diphenyl-tetrazolium bromide (MTT; Invitrogen, Carlsbad, CA, USA) were added to the cells per well, and then incubated at 37 °C for 4 h. A formazan pellet was dissolved in DMSO after removing the supernatant, and then the absorbance at 540 nm was measured using a spectrophotometer (Multiskan GO, Thermo Fisher Scientific, Vantaa, Finland). Cell viability was calculated using the following equation:

%Cell viability = [OD_sample_/OD_control_] × 100.

### 2.5. Plaque Assay

**Figure 1:**
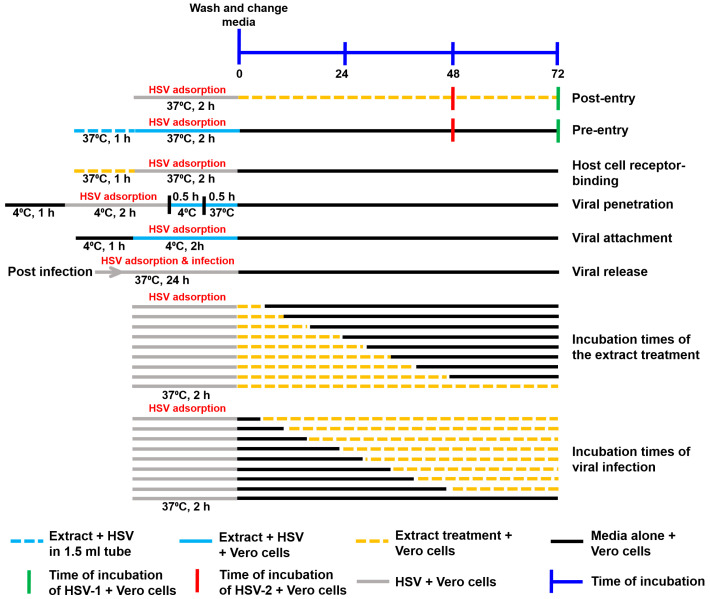
Schematic diagram of the performed experiments. The mechanism of action of the extracts against HSV, including the steps of post-entry, pre-entry, host cell receptor binding, viral penetration, viral attachment, viral release, different incubation times of extracts, and different incubation times of viral infection, were examined.

The assays of the extract’s mechanism against HSV are summarized in Figure 1.

#### 2.5.1. Post-Entry Step

Vero cells were seeded into a 24-well plate at 10^5^ cells/well and cultured in a complete medium for 24 h. The virus at multiplicity of infection (MOI) at 0.002 was infected in the cells for 2 h at 37 °C. After washing the cells to remove the unbound virus, each extract at various concentrations was prepared in a fresh medium containing 0.4% carboxymethyl cellulose (CMC), which was subjected to the cells and continuously cultured for 48–72 h. The cytopathic effect was observed under the microscope, and then the cells were fixed and stained with 10% formaldehyde and crystal violet. The number of plaques was counted by the naked eye. The percentage of viral inhibition was calculated using the following equations:

%Infection = [Number of plaques of treated cells/Number of plaque of control cells] × 100

%Inhibition = 100 − [%Infection]

#### 2.5.2. Pre-Entry Step

Vero cells were seeded into a 24-well plate at 10^5^ cells/well and maintained in a complete medium for 24 h. The virus at MOI 0.002 was mixed with each extract in the medium without FBS and then incubated for 1 h at 37 °C. The mixture was subjected to the cells and then incubated for 2 h. After removing the unbound virus, a fresh complete medium containing 0.4% CMC was added to the cells and continuously maintained for 48–72 h. The number of plaques was counted after crystal violet staining and used to calculate the percentage of viral inhibition as mentioned above.

#### 2.5.3. Host Cell Receptor-Binding Assay

Vero cells were seeded into a 24-well plate at a density of 10^5^ cells/well and incubated for 24 h. After removing the culture medium, the extracts at 100 μg/mL were subjected to the cells and incubated at 37 °C for an hour. Then, the extracts were removed and washed. The virus was loaded into the cells and incubated for 2 h at 37 °C. After removing unbound viruses and washing the cells, a complete medium containing 0.4% CMC was loaded into the cells. Next, the cells were continuously incubated for 72 h. The number of plaques was counted after crystal violet staining and used to calculate the percentage of viral inhibition as mentioned above.

#### 2.5.4. Viral Penetration

Vero cells were seeded into a 24-well plate at a density of 10^5^ cells/well and incubated for 24 h. The cells were then pre-incubated at 4 °C for an hour. HSV-1 dxpIII at MOI 0.002 was added to the cells and incubated at 4 °C for 2 h. After washing with cold-PBS, the extracts were subjected to the cells and continuously incubated at 4 °C for 30 min, and then immediately incubated at 37 °C for 30 min. To remove remaining unbound and adsorbed viruses, cells were washed with PBS at pH 3.0 and pH 11.0 for 10 s, respectively. The cells were washed with PBS pH 7.4 three times. A complete medium containing 0.4% CMC was loaded into the cells. Next, the cells were continuously incubated for 72 h. The number of plaques was counted after crystal violet staining and used to calculate the percentage of viral inhibition as mentioned above.

#### 2.5.5. Viral Attachment

Vero cells were seeded into a 24-well plate at a density of 10^5^ cells/well and incubated for 24 h. The cells were then pre-incubated at 4 °C for an hour. HSV-1 dxpIII at MOI 0.004 mixed with each extract in a ratio of 1:1 was added to the cells and incubated at 4 °C for 2 h. Cells were washed with PBS and then cultured in a complete medium containing 0.4% CMC. Next, the cells were continuously incubated for 72 h. The number of plaques was counted after crystal violet staining and used to calculate the percentage of viral inhibition as mentioned above.

#### 2.5.6. Viral Release Assay

The extracts were treated according to the experiment in the post-entry step, except for the medium used without CMC. The medium from the post-entry step was subjected to Vero cells and continuously incubated for 24 h. A complete medium containing 0.4% CMC was added to the cells after removing the viruses, and then maintained for 72 h. Plaque formation was observed after staining with crystal violet.

#### 2.5.7. Incubation Times of the Extract Treatment

Vero cells were seeded into a 24-well plate at a density of 10^5^ cells/well and incubated for 24 h. HSV-1 dxpIII at MOI 0.002 was added to the cells and incubated at 37 °C for 2 h. Each extract prepared in a fresh complete medium containing 0.4% CMC, which was added to the cells and incubated for 0, 6, 12, 18, 24, 30, 36, 48, and 72 h, and then replaced with a complete medium containing 0.4% CMC which was continuously incubated for 72, 66, 60, 54, 48, 42, 36, 24, and 0 h at 37 °C, respectively. Plaque was counted to determine the percentage of inhibition.

#### 2.5.8. Incubation Times of Viral Infection

Vero cells were seeded into a 24-well plate at a density of 10^5^ cells/well and incubated for 24 h. HSV-1 dxpIII at MOI 0.002 was added to the cells and incubated at 37 °C for 2 h. A complete medium containing 0.4% CMC was added to the cells and incubated for 0, 6, 12, 18, 24, 30, 36, 48, and 72 h, and then replaced with the extract prepared in a fresh medium containing 0.4% CMC and continuously incubated for 72, 66, 60, 54, 48, 42, 36, 24, and 0 h at 37 °C, respectively. Plaque was counted to determine the percentage of inhibition.

### 2.6. Evaluation of Viral ICP4, UL30, and gD and Cellular IL1B, IL6, STAT3, and NFKB1 mRNA Expression

Vero cells were infected with HSV at an MOI of 0.002 for 2 h. The cells were washed with PBS to remove unbound viruses and then replaced with a complete medium and the extracts at various concentrations, followed by continuous incubating for different time points. The cells were harvested and lysed in Trizol reagent (Invitrogen, Carlsbad, CA, USA) according to the manufacturer to isolate RNA and protein. After homogenizing the sample with Trizol and chloroform, and centrifuging at 12,000× *g* for 15 min, the upper phase was collected to precipitate RNA with isopropanol. The RNA pellet was separated using centrifugation at 12,000× *g* for 15 min at 4 °C, then washed with 75% ethanol and dissolved in 50 μL RNase-free water. The quantification and qualification of RNA were measured using a nanophotometer (Implen GmbH, Munich, Germany). The synthesis of cDNA was performed using a Revert Aid First Strand cDNA Synthesis Kit (Thermo Fisher Scientific, Waltham, MA, USA). The cDNA was diluted at 1:5 and then used as a template in an RT-PCR master mix (Bio-Rad, Hercules, CA, USA) with each internal control, *GAPDH*, viral *ICP4*, *UL30*, and *gD*, and cellular *IL1B*, *IL6*, *STAT3*, and *NFKB1* (Table S1). The amplification was run in the Eco48 real-time qPCR system (PCRmax, Staffordshire, UK) with conditions of 95 °C for 20 s followed by 60 °C for 30 s in 45 cycles. The expression level was measured using the relative quantification by calculating 2^−ΔΔCT^.

### 2.7. HSV-1 gB Protein Expression Detection by Western Blotting Analysis

Proteins were isolated from the phenolic phase (lower layer) after removing the upper phase from the mixture. Briefly, the phenol–ethanol supernatant was centrifuged to precipitate DNA. Protein was precipitated by additional isopropanol and then centrifuged at 7500× *g* for 5 min at 4 °C. After removing the supernatant, the protein pellet was washed twice with 0.3 M guanidine hydrochloride in 95% ethanol and incubated at room temperature for 20 min, and centrifuged at 7500 rpm for 5 min at 4 °C. The pellet was washed twice with 95% ethanol. The pellet was added with absolute ethanol following incubation for 20 min at room temperature and centrifugation at 7500× *g* for 5 min at 4 °C. Finally, the protein pellet was dried at room temperature for 20 min and dissolved in rehydration buffer (8M urea and 2% CHAPS). The proteins were separated by size in 12% Sodium Dodecyl Sulphate-Polyacrylamide Gel Electrophoresis and transferred into the nitrocellulose membrane. After transferring, the blotted membrane was blocked in PBS containing 5% skimmed milk and 0.1% Tween 20 for 1 h with shaking. The primary monoclonal beta-actin (1:1000 dilution; clone C4, sc-47778; Santa Cruz Biotechnology, Inc; Santa Cruz, CA, USA), and polyclonal HSV gB (1:1000 dilution, clone R69, kindly provided by Prof. Gary H. Cohen and Prof. Roselyn J. Eisenberg, University of Pennsylvania, Philadelphia, PA, USA) antibody was loaded onto the membrane and incubated in a refrigerator overnight. Then, the membrane was washed with PBS containing 0.1% Tween 20 three times. The secondary antibodies—horseradish peroxidase-conjugated goat anti-rabbit immunoglobulin G (Cat. no. G21234, Invitrogen, San Francisco, CA, USA) (1:5000) for gB antibody, and IgGκ binding protein-HRP (m-IgGκ BP-HRP) (sc-516102; Santa Cruz Biotechnology) for beta-actin antibody—were added to the membrane and incubated for 2 h. After washing, the proteins blotted in the membrane were detected using a chemiluminescence imaging system (UVITech, Cambridge, UK). The intensity of gB and the beta-actin proteins was measured using ImageJ version 1.51j8 software (Wayne Rasband, National Institutes of Health, Bethesda, MD, USA).

### 2.8. Phytochemical Profile Analysis Using Liquid Chromatography–Tandem Mass Spectrometry (LC–MS/MS)

The extract was dissolved in methanol and 0.2% formic acid/water before being subjected to LC–MS/MS analysis to determine the total ion intensity of the identified compounds. Raw data were processed with Compound Discoverer software, version 3.1 (Thermo Fisher Scientific, Waltham, MA, USA), to identify phytochemicals. The data of peak identification, alignment, and feature extraction were analyzed in positive mode. The retention time (RT) and mass-to-charge ratio (*m*/*z*) were determined according to a retention time deviation of 0.5 min and a mass deviation of 5 ppm. The peak extraction was then performed according to the following conditions: 5 ppm of mass deviation, 30% of signal strength deviation, signal-to-noise ratio of 2, and fine isotopic pattern matching > 90% of the precursor and the characteristic product ions. The quantification of the peak area was measured. The target *m*/*z* ions were predicted as a molecular formula and compared to the mzCloud and ChemSpider online databases. The structural elucidation and transformations were indicated for each chromatographic peak by the Fragment Ion Search^TM^ (FISh) function. The FISh coverage score was calculated, and fragments on the MS/MS spectrum were annotated with molecular weight, structure, and elemental composition. The highest MS/MS coverage scores were selected for annotation.

### 2.9. Molecular Docking Analysis and Potential Drug Target Prediction of 2-Methoxy-9H-xanthen-9-one, Isorhapontigeninfound, Piperine, Pellitorine, Sargassopenilline D, and Parmoether A

Crystal structures of HSV DNA polymerase (8V1T) were retrieved from the Protein Data Bank (https://www.rcsb.org/ (accessed on 5 February 2025). The structures 2-Methoxy-9H-xanthen-9-one (CID: 71034), isorhapontigenin (CID: 5318650), piperine (CID: 638024), and pellitorine (CID: 5318516) were downloaded from NCBI PubChem (https://pubchem.ncbi.nlm.nih.gov/, accessed on 3 February 2025). Sargassopenilline D and parmoether A were downloaded from COCONUT 2.0 (https://coconut.naturalproducts.net/compounds/CNP0138706.1, accessed on 3 February 2025) and ChEBI (https://www.ebi.ac.uk/chebi/chebiOntology.do?chebiId=CHEBI:205895, accessed on 3 February 2025), respectively. The ligand and receptor were prepared before docking using the BIOVIA Discovery Studio Visualizer (v21.1.0.20298) and AutoDock Tool (v1.5.7 Dec_19_18), including by removing water, adding hydrogen, assigning charges, and generating molecular surfaces. Docking simulations were performed using the AutoDock Tool Vina [22]. The docking pose with the lowest binding energy and minimum root mean square deviation (RMSD) and highest binding energy (kcal/mol) was considered as the most suitable. The PyMOL Molecular Graphics System (Version 2.5.7, Schrödinger, LLC) and BIOVIA Discovery Studio Visualizer software (v21.1.0.20298) were used to visualize the ligand and receptor interaction in 3D and 2D structures, respectively. To determine the 6 candidate compounds that possess favorable absorption, distribution, metabolism, and excretion properties, the online tool SwissADME software (http://www.swissadme.ch, accessed on 28 February 2025) was used. The drug-likeness of the compounds was assessed according to Lipinski’s rule of five and Veber’s rule [23,24].

### 2.10. Statistical Analysis

The data were expressed as the mean ± standard error of the mean (SEM). The different levels were analyzed using one-way ANOVA in GraphPad Prism 8 software (version 8.0.2, GraphPad Software Inc., La Jolla, CA, USA). Significant differences were indicated as * (*p* < 0.05), ** (*p* < 0.01), and *** (*p* < 0.001).

## 3. Results

### 3.1. Cytotoxicity of Kerra^TM^, KS^TM^, and Minoza^TM^ in Vero Cells

The 50% cytotoxic concentrations (${\mathrm{CC}}_{50}$) of ACV, Kerra^TM^, KS^TM^, and Minoza^TM^ are shown in Table 1. KS^TM^ had the highest cytotoxicity, followed by Kerra^TM^ and Minoza^TM^, respectively.

### 3.2. Kerra^TM^, KS^TM^, and Minoza^TM^ Significantly Inhibited the Infection with HSV-1 and HSV-2 Wild-Type Strains in the Post-Entry Step

**Figure 2:**
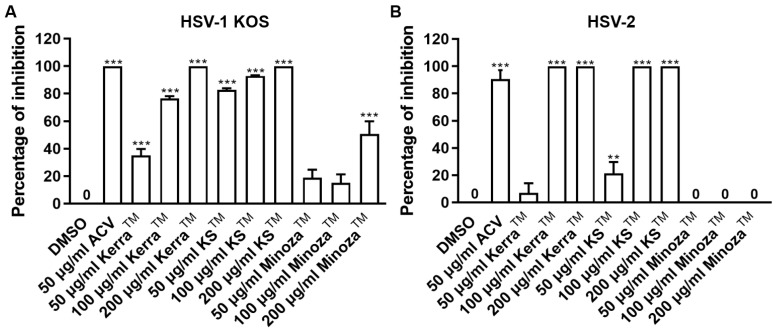
Kerra^TM^-, KS^TM^-, and Minoza^TM^-treated Vero cells infected with HSV-1 KOS and HSV-2 in the post-entry step. Each of (**A**) HSV-1 and (**B**) HSV-2 at MOI 0.002 was infected in Vero cells for 2 h before the extract was added at 50, 100, and 200 μg/mL for 48–72 h. Plaque was counted to determine the percentage of inhibition. The symbols ** and *** indicate significant differences (*p* ˂ 0.01 and 0.001, respectively) between the extract and DMSO-treated cells. Bar charts represent the mean and SEM of triplicate experiments.

Treatment of herpes labialis is provided after the patient has already been infected with HSV, which is termed the post-infection or post-entry step. Therefore, to investigate the ability of each extract to reduce plaque formation after viral internalization, each extract was treated with wild-type strains of HSV-1- and HSV-2-infected cells. If the extract could reduce the number of plaques, it might target various mechanisms and not only bind directly to viral molecules. Based on the results, all extracts (particularly 200 μg/mL) significantly inhibited the infection with HSV-1 KOS at 100% inhibition of plaque formation in the post-entry step in a dose-dependent manner, as shown in Figure 2A and Figure S1A. In addition, Kerra^TM^ and KS^TM^ at 100–200 μg/mL inhibited HSV-2 infection (100% inhibition, but not Minoza^TM^), as shown in Figure 2B and Figure S1B. Notably, at 50 μg/mL, KS^TM^ produced the highest inhibition of HSV-1 KOS (82.65% ± 1.24%) and HSV-2 (21.48% ± 8.19%) compared to Kerra^TM^ and Minoza^TM^. These results demonstrated that Kerra^TM^ and KS^TM^ could be potential drugs with antiviral activity for wild-type strains HSV-1 and HSV-2.

### 3.3. Kerra^TM^, KS^TM^, and Minoza^TM^ Significantly Inhibited HSV-1 dxpIII in the Post-Entry Step

**Figure 3:**
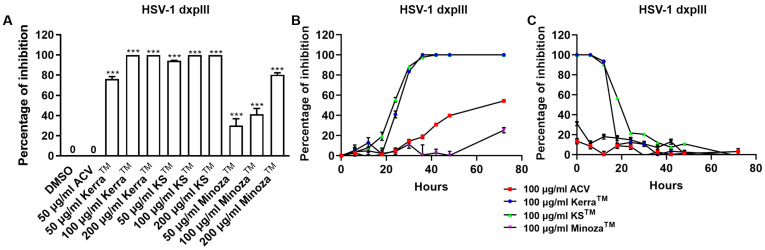
Kerra^TM^-, KS^TM^-, and Minoza^TM^-treated Vero cells infected with HSV-1 dxpIII in the post-entry step. (**A**) HSV-1 dxpIII at MOI 0.002 was infected in Vero cells for 2 h before the extract was added for 72 h. (**B**) The incubation time of the extract treatment in HSV-1 dxpIII-infected Vero cells was determined in each 100 μg/mL of Kerra^TM^, KS^TM^, and Minoza^TM^ treatment for 0, 6, 12, 18, 24, 30, 36, 48, and 72 h after removing unbound viruses incubated for 2 h. (**C**) The incubation time of viral infection was investigated in HSV-1 dxpIII-infected Vero cells for 0, 6, 12, 18, 24, 30, 36, 48, and 72 h which were then replaced with 100 μg/mL each of Kerra^TM^, KS^TM^, and Minoza^TM^ for 72, 66, 60, 54, 48, 42, 36, 24, and 0 h, respectively. Plaque was counted to determine the percentage of inhibition. The symbol *** indicates significant differences (*p* ˂ 0.001) between the extract and DMSO-treated cells. Bar charts represent the mean and SEM of triplicate experiments.

To compare the inhibitory effect of the three extracts and ACV, HSV-1 dxpIII was infected in Vero cells and then treated with the extracts or ACV. Unexpectedly, all three extracts significantly reduced plaque formation of HSV-1 dxpIII; in particular, KS^TM^ (94.29% ± 3.33–100.00% ± 0.00% inhibition of 50–200 μg/mL, respectively) produced the highest %Inhibition, followed by Kerra^TM^ (76.37% ± 2.25–100.00% ± 0.00% inhibition of 50–200 μg/mL, respectively) and Minoza^TM^ (30.15% ± 6.66–80.48% ± 1.86% inhibition of 50–200 μg/mL, respectively), whereas 50 μg/mL ACV could not inhibit viral infection (Figure 3A and Figure S2). The extracts were further investigated to elucidate the different incubation times of the extract treatment exerting HSV-1 dxpIII inhibition. The result showed that 100 μg/mL Kerra^TM^ and KS^TM^ dramatically reduced viral plaque formation after 18 h of incubation time, whereas 100 μg/mL ACV and Minoza^TM^ gradually increased %Inhibition more slowly than either extract (Figure 3B). When considering the incubation time for viral infection, 100 μg/mL Kerra^TM^ and KS^TM^ dramatically increased %Inhibition (>50%) within 20 h incubation time for viral infection; in particular, KS^TM^ inhibited plaque formation within 24 h while ACV and Minoza^TM^ increased %Inhibition by less than 40% at 0 h (Figure 3C). Interestingly, both Kerra^TM^ and KS^TM^ extracts dramatically reduced plaque formation at a high MOI of 0.02–2.0 (approximately 10^3^–10^5^ PFU/well, respectively) of HSV-1 dxpIII infection compared to DMSO treatment (Figure S3). This study demonstrated that KS^TM^ showed the highest effective suppression of HSV-1 dxpIII, followed by Kerra^TM^.

### 3.4. Inhibitory Concentrations and Selective Index of Kerra^TM^, KS^TM^, and Minoza^TM^ on Anti-HSV-1 KOS, HSV-1 dxpIII, and HSV-2 in Vero Cells

Table 2 indicates the abilities of Kerra^TM^, KS^TM^, and Minoza^TM^ in relation to anti-HSV-1 KOS, HSV-1 dxpIII, and HSV-2 infection. The 50% inhibitory concentrations (${\mathrm{IC}}_{50}$) of KS^TM^ and Kerra^TM^ were lower than for Minoza^TM^ in all three HSV strains, even though Minoza^TM^ produced the highest score of SI in HSV-1 dxpIII (159.669). Based on the ${\mathrm{IC}}_{50}$ and SI values, KS^TM^ and Kerra^TM^ were the most efficient drugs and safe for HSV-1 KOS, HSV-1 dxpIII, and HSV-2 treatments.

### 3.5. Kerra^TM^, KS^TM^, and Minoza^TM^ Inhibited HSV-1 KOS, dxpIII, and HSV-2 Infection in the Pre-Entry Step

**Figure 4:**
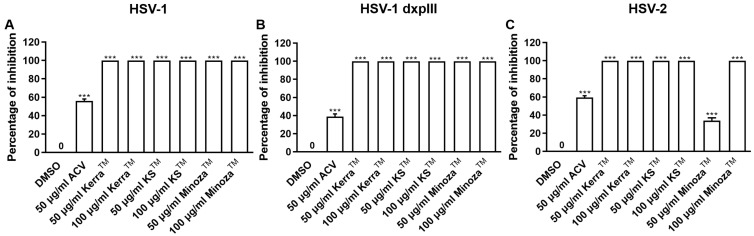
Kerra^TM^-, KS^TM^-, and Minoza^TM^-treated Vero cells infected with HSV-1 KOS, HSV-1 dxpIII, and HSV-2 in the pre-entry step. Each of (**A**) HSV-1 KOS, (**B**) HSV-1 dxpIII, and (**C**) HSV-2 at MOI 0.002 was mixed with Kerra^TM^, KS^TM^, or Minoza^TM^ at 50 and 100 μg/mL for 1 h before infection in Vero cells for 2 h. After removing unbound viruses, the cells were maintained in a complete medium for 48–72 h post-infection. Plaque was counted to determine the percentage of inhibition. The symbol *** indicates a significant difference (*p* ˂ 0.001) between the extract and DMSO-treated cells. Bar charts represent the mean and SEM of triplicate experiments.

To explore the mechanism of the three extracts in the pre-entry step, each extract was pre-incubated with HSV-1 KOS, dxpIII, and HSV-2. Based on the results, Kerra^TM^ and KS^TM^ at 50 and 100 μg/mL (but not Minoza^TM^) completely inhibited plaque formation (100% inhibition) from HSV-1 KOS, HSV-1 dxpIII, and HSV-2 infection in the pre-entry step (Figure 4 and Figure S4). As mentioned above, Kerra^TM^ and KS^TM^ at 50 μg/mL had higher effective suppression of plaque formation in HSV-1 dxpIII than ACV in the post-entry step, demonstrating their potential application as drugs for the treatment of the HSV ACV-resistant strain. Therefore, the mode of action of extracts needed to be clarified, including the host cell receptor-binding, viral penetration, viral attachment, and viral release assay. Figure S5A shows the results of all three extracts and ACV that could not inhibit plaque formation in the host cell receptor-binding step. Simultaneously, Kerra^TM^ and KS^TM^ at 100 μg/mL prevented HSV-1 dxpIII plaque formation in the viral penetration, viral attachment and viral release assay (Figure S5B–D). From these results, we demonstrated that the steps of pre-entry, viral penetration, viral attachment, prevention of viral release, and blocking viral replication inside the cells were the main mechanisms of Kerra^TM^ and KS^TM^ in anti-HSV infection.

### 3.6. Effect of Kerra^TM^, KS^TM^, and Minoza^TM^ Treatment in HSV-Infected Vero Cells on Viral gD and Cellular IL1B, IL6, STAT3, and NFKB1 mRNA Expression

**Figure 5:**
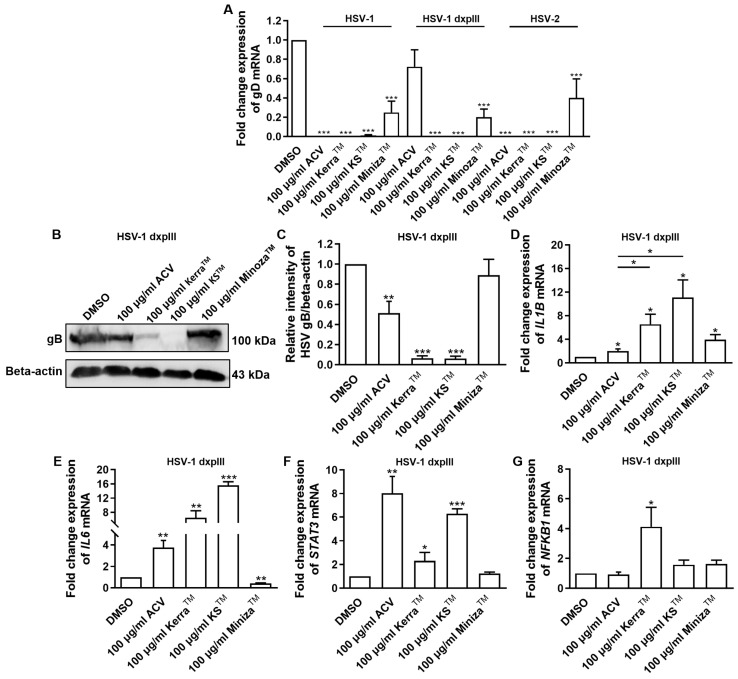
HSV gB, *gD*, *IL1B*, *IL6*, *STAT3*, and *NFKB1* expression in Kerra^TM^-, KS^TM^-, and Minoza^TM^-treated Vero cells. (**A**) mRNA expression levels of HSV-1 KOS, HSV-1 dxpIII, and HSV-2 *gD* were investigated in 50–200 μg/mL Kerra^TM^-, KS^TM^-, and Minoza^TM^-treated Vero cells for 48 h. (**B**,**C**) Viral glycoprotein gB levels in ACV, DMSO, Kerra^TM^, KS^TM^, and Minoza^TM^ treatments were determined using Western blot analysis. Cellular (**D**) *IL1B*, (**E**) *IL6*, (**F**) *STAT3*, and (**G**) *NFKB1* were determined in HSV-1 dxpIII-infected Vero cells. DMSO and ACV acted as negative and positive controls, respectively. The symbols *, **, and *** indicate significant differences (*p* < 0.05, 0.01, and 0.001, respectively). Bar charts represent the mean and SEM of triplicate experiments.

All extracts significantly reduced the expression of *gD* of HSV-1 KOS, dxpIII, and HSV-2, which is the gene encoding the receptor-binding glycoprotein that indicated the reduction in virion production by the Kerra^TM^, KS^TM^, and Minoza^TM^ treatments (Figure 5A). In addition, gB structural protein was downregulated in Kerra^TM^- and KS^TM^-treated cells (Figure 5B,C), corresponding to the viral *gD* mRNA level. These data indicated that the extracts, particularly Kerra^TM^ and KS^TM^, effectively reduced viral glycoprotein in both mRNA and protein levels. Because of the higher efficiency of all Kerra^TM^, KS^TM^, and Minoza^TM^ treatments at reducing the number of plaques than ACV in the HSV-1 dxpIII-infected cells, the biological regulation of these extracts in gene-related immunity was investigated.

The mRNA levels of cellular genes that play a role in antiviral infection were determined, including *IFNa1*, *NFKB1*, *IL1B*, *IL6*, and *STAT3* [25,26,27,28]. Notably, ACV, Kerra^TM^, KS^TM^, and Minoza^TM^ upregulated *IL1B* mRNA expression, whereas ACV, Kerra^TM^, and KS^TM^ (but not Minoza^TM^) increased *IL6* and *STAT3* (Figure 5D–F). NF-κB encoded by *NFKB1* upstream of the *IL1*, *IL6*, and *STAT3* genes was considerably increased in the Kerra^TM^ treatment, whereas it was only slightly elevated in the KS^TM^- and Minoza^TM^-treated cells. Unfortunately, the mRNA expression of *IFNa1* in all groups could not be detected (Ct > 45) in the 80 ng cDNA/reaction. The possible mechanism of Kerra^TM^ and KS^TM^ is the upregulation of transcriptional factor NF-κB, resulting in increased *IL1B*, *IL6*, and *STAT3* expression, whereas Minoza^TM^ upregulated *IL1B* partially via NF-κB, as well as via another pathway.

### 3.7. Effect of Kerra^TM^ and KS^TM^ on Immediate Early Gene ICP4, Early Gene UL30, IL1B, and NFKB1 at Different Time Points

**Figure 6:**
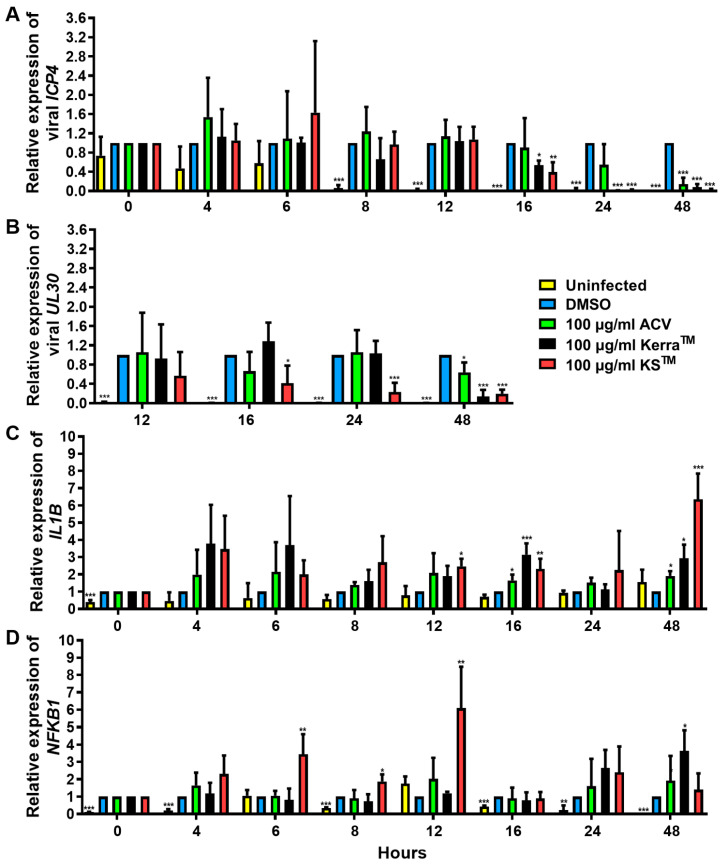
*ICP4*, *UL30*, *IL1B,* and *NFKB1* expression in Kerra^TM^- and KS^TM^-treated Vero cells. The mRNA expression levels of HSV-1 dxpIII (**A**) *ICP4*, (**B**) *UL30*, and cellular (**C**) *IL1B* and (**D**) *NFKB1* were investigated in 100 μg/mL Kerra^TM^-, and KS^TM^-treated Vero cells for 0–48 h. DMSO and ACV acted as negative and positive controls, respectively. The symbols *, **, and *** indicate significant differences (*p* < 0.05, 0.01, and 0.001, respectively). Bar charts represent the mean and SEM of triplicate experiments.

To elucidate the effect of Kerra^TM^ and KS^TM^ on the viral life cycle as well as antiviral immunity, viral IE *ICP4*, E *UL30*, *IL1B*, and *NFKB1* mRNA expression in HSV-1 dxpIII-infected Vero were determined 0–48 h after incubation in the extract treatment. Kerra^TM^ and KS^TM^ significantly reduced *ICP4* at 16 h, whereas only KS^TM^ significantly decreased *UL30* at this time point, but Kerra^TM^ exhibited this viral gene expression at 24 h. However, *ICP4* and *UL30* were dramatically expressed at 8 and 12 h post-infection compared with non-infected cells (Figure 6A,B). Therefore, both extracts may not fully directly impact *ICP4* and *UL30* downregulation. Viral infection alone at 0–16 h after viral adsorption tended to increase *IL1B* mRNA levels, but they were decreased at 48 h (Figure 6C). Interestingly, Kerra^TM^ and KS^TM^ increased *IL1B* expression at 4–48 h compared with uninfected and infected control cells (Figure 6C). Simultaneously, KS^TM^ gradually increased and decreased *NFKB1* at 4–12 h and 16–48 h, respectively. Kerra^TM^ treatment elevated this mRNA level at 24–48 h (Figure 6D). To elucidate the effect of KS^TM^ in HSV-1 dxpIII-infected HeLa, the expression of *IFNa1* and *IL1B* was investigated at different time points. KS^TM^ significantly induced *IFNa1* at 24 h and gradually decreased at 48 h (Figure S6A). In addition, this extract upregulated *IL1B* at 12–48 h, corresponding to the result in HSV-1 dxpIII-infected Vero cells (Figure S6B). This result may indicate the mechanism by which both extracts indirectly downregulate the IE gene, resulting in the expression levels of E and L genes and increasing *IL1B* and *NFKB1* mRNA expression.

### 3.8. Phytochemical Profiling and Qualitative Metabolite Analysis

Table 3 represents the phytochemicals in each of Kerra^TM^, KS^TM^, and Minoza^TM^. A total of 414 and 378 annotated phytochemical formulas are found in Kerra^TM^ and Minoza^TM^ (Table S2), respectively, using LC-MS/MS [29].

### 3.9. Docking Results for Candidate Most Abundant Phytochemicals in Kerra^TM^, KS^TM^, and Minoza^TM^

**Figure 7:**
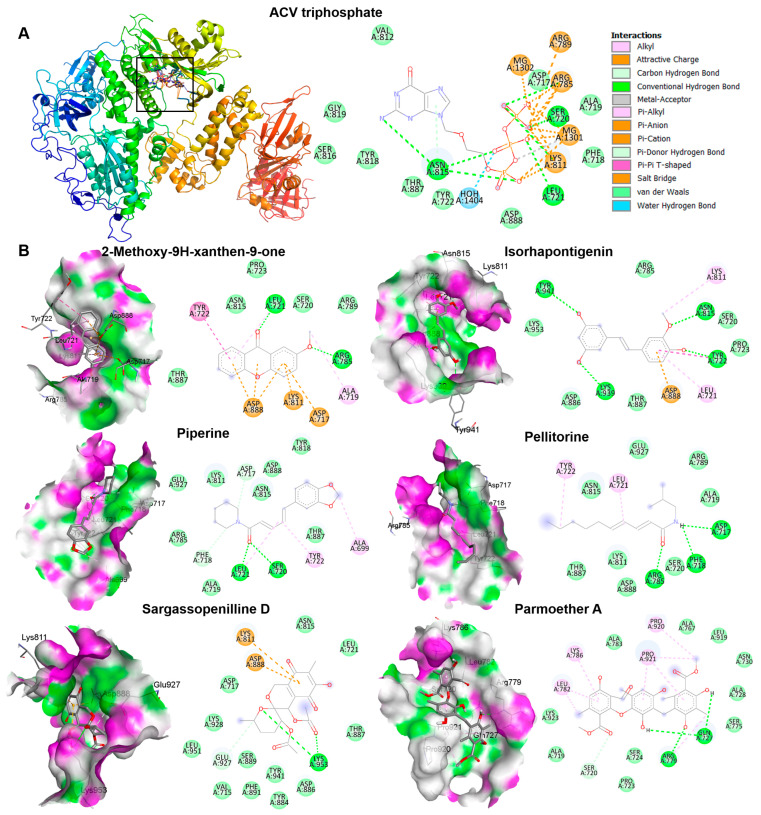
The interactions of HSV-1 DNA polymerase when docked with candidate compounds. (**A**) The docking poses of ACV triphosphate and HSV-1 DNA polymerase are shown in the 3D and 2D structures. The small boxes represent the active site binding pocket of HSV-1 DNA polymerase and ACV interaction. The grid was fixed in the active site (x = 147.2865, y = 145.1162, and z = 123.7358 Å) to analyze the interaction between ligand and receptor. (**B**) The 3D and 2D structures indicate the interaction between HSV-1 DNA polymerase and each compound, 2-Methoxy-9H-xanthen-9-one, isorhapontigenin, piperine, pellitorine, sargassopenilline D, and parmoether A.

The candidate phytochemicals 2-Methoxy-9H-xanthen-9-one, isorhapontigeninfound, piperine, pellitorine, sargassopenilline D, and parmoether A found in Kerra^TM^, KS^TM^, and Minoza^TM^ were collected to investigate their interactions on HSV-1 DNA polymerase, which is the target of ACV triphosphate. The results indicate that all of these six compounds could bind to HSV-1 DNA polymerase in the same active site of ACV triphosphate, as shown in Figure 7. To evaluate the drug-likeness and predict a novel compound against HSV-1, the criteria of Lipinski’s rule of five and Verber’s rules are considered using SwissADME predictor. The five candidate compounds have desirable drug-like properties, except parmoether A (Table 4). Based on docking analysis and drug-likeness prediction, 2-Methoxy-9H-xanthen-9-one, piperine, and sargassopenilline D found in Kerra^TM^, KS^TM^, and Minoza^TM^ show high binding energy closely resembling ACV, and are considered to have drug-like characteristics. Therefore, Kerra^TM^, KS^TM^, and Minoza^TM^ could be alternative drugs for HSV-associated disease, particularly drug-resistant strains.

## 4. Discussion

Three Thai traditional medicines, namely Kerra^TM^, KS^TM^, and Minoza^TM^ (the trademark names of the products), showed potential as agents for anti-HSV infection. Even though Kerra^TM^, KS^TM^, and Minoza^TM^ less efficiently inhibited the wild-type strains of HSV-1 and HSV-2 than ACV, all three extracts significantly inhibited HSV-1 dxpIII, particularly Kerra^TM^ and KS^TM^, which were more effective in viral suppression than this standard drug. The lower inhibitory activity of Minoza^TM^ may be involved in the low amount of different types of promising bioactive compounds against HSV (Table 3 and Table S2). However, 400 and 800 μg/mL Minoza^TM^ increased %Inhibition of plaque formation (91.29% ± 1.49% and 100% ± 0.00%) (Figure S7).

Considering the interaction between the extracts and viral molecules, the pre-entry step (the process of determining their interaction before viral binding to the receptor) indicated the highest efficiency of plaque inhibition in the Kerra^TM^, KS^TM^, and Minoza^TM^ treatments compared with the post-entry step. Therefore, the extracts may directly bind to viral glycoprotein gB, gC, gH, gK, gL, or gL, particularly gD, which binds to the major receptors including herpesvirus entry mediator, Nectin1, Nectin2, and 3-O-sulfated heparan sulfate proteoglycan, resulting in the inhibition of viral attachment and infection [32]. In addition, these potential drugs (Kerra^TM^, KS^TM^, and Minoza^TM^) have been investigated in human papillomavirus type 16 (HPV16) in vitro; these three extracts significantly inhibited HPV16 pseudovirus infection, which may also indicate the ability of these extracts to interact with L1/L2 HPV16 directly [21].

**Figure 8:**
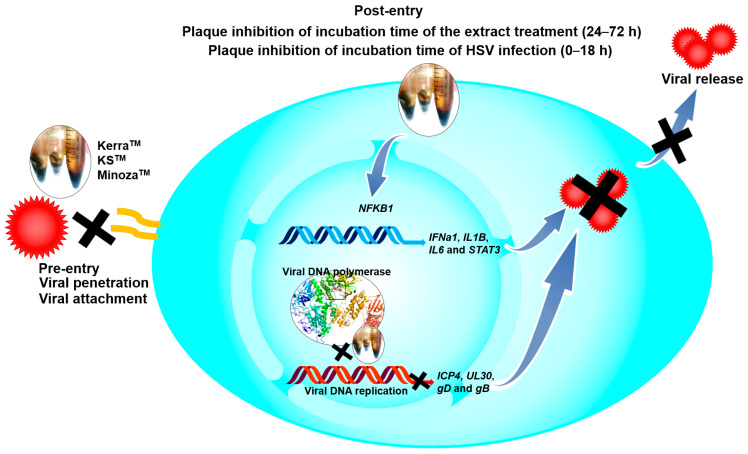
The possible mechanisms of Kerra^TM^, KS^TM^, and Minoza^TM^ on anti-HSV.

Notably, KS^TM^ most effectively inhibited HSV-1 dxpIII, followed by Kerra^TM^, both of which showed higher ability against this viral strain than ACV, even though this virus is a phosphonoacetic acid- and phosphonoformate-resistant strain [12]. Both phosphonoacetic acid and phosphonoformate (foscarnet) are pyrophosphosphate analogues that directly bind to the viral pyrophosphate binding site in the DNA polymerase active site, resulting in the inhibition of the exchange of pyrophosphate from deoxynucleoside triphosphate for DNA polymerization [33,34]. Simultaneously, ACV is an analogue of the nucleoside deoxyguanosine that inhibits the next required nucleotide linkage in the viral DNA polymerase active site through ACV monophosphate being catalyzed by viral thymidine kinase and then converted to the triphosphate form by cellular enzymes [35]. These drugs provide competitive activity against HSV [34,35]. The mechanism of HSV resistance to ACV and phosphonoformate has been reported through the mutation of viral thymidine kinase and DNA polymerase [36,37]. Even though HSV-1 dxpIII is not commonly used as an ACV-resistant strain, this strain is resistant to ACV compared with the results in HSV-1 KOS and HSV-2 in our findings. Another study demonstrated that the ${\mathrm{IC}}_{50}$ and SI values of ACV treatment on anti-HSV-1 dxpIII were 161.45 μM (36.36 μg/mL) and >39.64, respectively, and it produced lower efficient inhibition than the ACV treatment of HSV-1 KOS [12]. Kerra^TM^ and KS^TM^ at ${\mathrm{IC}}_{50}$ levels more effectively inhibited HSV-1 KOS, HSV-1 dxpIII, and HSV-2 (Table 2). In addition, both extracts considerably reduced the viral *gD* mRNA and gB protein levels compared to Minoza^TM^ (Figure 5). The reduction in *gD* and gB expression in Kerra^TM^- and KS^TM^-treated cells may be due to their ability to inhibit HSV multiplication through the regulation of the viral immediate early (IE) gene, including HSV transcription factors ICP0 and ICP4, resulting in the reduction in the early gene involvement in viral replication. Subsequently, the expression of late genes, including gD and gB, is decreased [20,38]. In the present study, Kerra^TM^ and KS^TM^ did not suppress *ICP4* and *UL30* within 8 and 12 h of incubation, but significantly suppressed them in 16 and 24 h, respectively, indicating that both extracts may not directly inhibit viral IE and E expression (Figure 8).

The IL-1 signaling cascade regulates many genes to promote the inhibition of viral infection in human skin, as well as activating NF-κB translocation to the nucleus via the IL-1 receptor (IL-1R) [26]. Additionally, NF-κB encoded by the *NFKB1* gene is a key transcriptional factor of many cytokines, including IL-1 and IL-6, to control innate and adaptive immunity, especially the antiviral innate response [39,40,41]. Notably, HSV-1 suppressed NF-κB function, contributing to the elevation of viral production in infected human monocyte cell line U937, while NF-κB activation limited viral replication. Other studies demonstrated that natural extracts could inhibit HSV-1 via IL-1 upregulation in human embryonal lung fibroblast cell line MRC-5 cells [17]. In addition, HSV-1 tegument protein VP22 inhibited AIM2 inflammasome activation, leading to decreased IL-1β secretion and then increased viral titers in an in vivo study [42]. Corresponding to other studies, Kerra^TM^ significantly increased both IL-1β (*IL1B*) and NF-κB (*NFKB1*) to promote HSV-1 inhibition in Vero cells. Simultaneously, KS^TM^ and Minoza^TM^ significantly increased *IL1B* but slightly increased *NFKB1*; this event may be involved in other mechanisms, including AIM2-dependent inflammasome activation, not only for *NFKB1*, that can promote *IL1B* expression [43]. IL-6 is a multifunctional cytokine well known as a proinflammatory cytokine; however, the lack of IL-6 increased the symptom severity, morbidity, and mortality of HSV infection [27,41]. STAT3 is a transcriptional factor that plays an important role in enhancing the efficiency of cell-mediated immunity against viral infectivity, including HSV [28]. Inhibition of STAT3 activation promotes HSV reactivation [44]. The effect of the Kerra^TM^ and KS^TM^ treatments upregulating *IL6* and *STAT3* may indicate their role in enhancing immunity to prevent or clear viral infection. IFNα belongs to the type I interferon produced in various kinds of cell types, including epithelial cells, and is induced by IRF3/7 transcriptional regulators; this induction appeared when HSV-1 was uncoated in host cytoplasm via IFI16 receptor signaling [45,46]. However, *IFNa1* mRNA expression was not determined in any of the extracts, acyclovir, or DMSO-treated Vero cells, which was consistent with another study [47]. Among these three extracts, KS^TM^, which was the most effective inhibitor of HSV, upregulated antiviral *IFNa1* in 24 h, and *IL1B* in 24 and 48 h in HeLa cells which could express this cellular gene. The upregulation of *IFNa1*, *IL1B*, *IL6*, *NFKB1*, and *STAT3* may contribute to antiviral defense but not side effects of extract-induced stress because of subtoxic concentrations of the extracts used in the experiments. These results may indicate that this mechanism may be involved in the induction of antiviral immunity (Figure 8).

Kerra^TM^ is a product comprising nine different medicinal plants—*Citrus aurantifolia* (Christm.) Swingle., *Combretum quadrangulare* Kurz., *Dracaena loureiri* Gagnep., *Dregea volubilis* Benth. ex Hook.f., *Momordica cochinchinensis* (Lour.) Spreng., *Schumannianthus dichotomus* (Roxb.) Gagnep., *Tarenna hoaensis* Pit., *Tiliacora triandra* Diels., and *Tinospora cordifolia*—containing several kinds of bioactive compounds such as phytochemicals, pterostilbene, coumarins, *O*-naphthoquinones, mansorin-A, mansorin-B, mansorin-C, mansorin II, mansorin-I, and mansonone-G [21,29]. These bioactive compounds can regulate biological consequences, including reactive oxygen species which play a role in HSV pathogenesis [48]. Notably, this product effectively inhibited not only HPV and HSV but also human immunodeficiency virus-1 reverse transcriptase, indicating its potential as a drug to treat antiviral infectious diseases [21,31].

KS^TM^ contains 20 different plants—*Acorus calamus* L., *Amomum cardamomum* L. (seed), *Anaxagorea luzonensis* A. Gray (wood), *Boesenbergia rotunda* (L.) Mansf., *Cinnamomum camphora* (L.) J.Presl., *Derris scandens* (Roxb.) Benth, *Ficus foveolata* Wall., *Mallotus repandus* (Willd.) Mull. Arg., *Myristica fragrans* Houtt., *Piper interruptum* Opiz, *Piper nigrum* L. (climber), *Piper nigrum* L. (seed).), *Piper retrofractum* Vahl (fruit), *Piper sarmentosum* Roxb., *Plumbago indica* L., *Senna garrettiana* (Craib), H.S.Irwin and Barneby, *Syzygium aromaticum* (L.) Merr. and L.M. Perry. (flower), *Zingiber cassumunar* Roxb., *Zingiber officinale* Roscoe, and *Zingiber zerumbet* (L.)—and contains several bioactive compounds, including triterpenoids, monoterpenoids, sesquiterpenoids, phenolics, alkaloids, steroids, aldehydes, ketones, alcohols, and esters [21,49,50]. Like the Kerra^TM^ treatment results, KS^TM^ may be a potential drug with antiviral and anti-cancer activities [21,51].

Minoza^TM^ is prepared from six different plant components—*Aloe vera* (L.) Burm. F., *Glycosmis pentaphylla* (Retz.) DC., *Murdannia loriformis* (Hassk.) R.S. and Kammathy, *Parinari anamensis* Hance., *Phlogacanthus sirindhorniae* (K.Larsen) Mackinder and R. Clark, and *Smilax corbularia* Kunth.—and includes many kinds of bioactive compounds, such as phenols, flavonoids, tannins, alkaloids, and steroids, while also producing the highest IC50 value in all three HSV strains (Table 2). Corresponding to another study, it had the lowest effect in anti-HPV and anti-cervical cancer [21].

The complex interactions between ligands and receptor targets and the prediction of drug-like characteristics were extensively used to elucidate drug-like parameters of the potential drugs or substances. As mentioned above, HSV DNA polymerase is a target of anti-HSV agents including ACV; therefore, to study the possible mechanisms of the extracts against HSV, six candidate phytochemicals found in Kerra^TM^, KS^TM^, and Minoza^TM^ were analyzed with this viral protein and compared with ACV. Among the six high-abundance candidate phytochemicals, 2-Methoxy-9H-xanthen-9-one, piperine, and sargassopenilline D in Kerra^TM^, KS^TM^, and Minoza^TM^, respectively, are the most promising drugs for HSV treatment, demonstrating high binding energy closely resembling ACV triphosphate (−7.1 kcal/mol) (Table 4). 2-Methoxy-9H-xanthen-9-one and isorhapontigenin found in Kerra^TM^ interacted with HSV-1 DNA polymerase on LEU721, TYR722, ARG785, ASN815, LYS939, and TYR941 residues with hydrogen bonds. Piperine and pellitorine detected in KS^TM^ interacted with LEU721 and SER720, and ASP717, PHE718, and ARG785 residues of this viral protein through hydrogen bonds with hydroxyl and carbonyl groups of compounds, respectively. Sargassopenilline D and parmoether A detected in Minoza^TM^ bound to ARG779, GLN727, and LYS953 with hydrogen bonds. These results suggest that the highly abundant phytochemicals found in the three extracts may directly interact with HSV DNA polymerase, leading to viral inhibition (Figure 8).

This study has revealed the effects of commercial herbal products against HSV through in vitro and in silico methods that cannot entirely explain the overall outcomes of their activity in humans. Therefore, the inhibitory effect of these extracts with or without the synergistic combination of a gold standard drug needs to be further elucidated through an in vivo study and in clinical trials to explore a novel drug for anti-HSV, particularly drug-resistant strains.

## 5. Conclusions

KS^TM^ and Kerra^TM^ were highly effective inhibitors against HSV-1 and HSV-2. Kerra^TM^, KS^TM^, and Minoza^TM^ may be used as potentially effective drugs to treat diseases associated with acyclovir-resistant HSV strains, including HSV-1 dxpIII.

## Figures and Tables

**Table 1:** ${\mathrm{CC}}_{50}$ values (mean ± SEM) of ACV, Kerra^TM^, KS^TM^, and Minoza^TM^ in Vero cells.

Extract	${\mathrm{CC}}_{50}$ (mg/mL)
ACV	7.545 ± 0.133
Kerra^TM^	2.846 ± 0.025
KS^TM^	2.349 ± 0.026
Minoza^TM^	11.670 ± 1.804

**Table 2:** ${\mathrm{IC}}_{50}$ (mean ± SEM) and SI values of Kerra^TM^, KS^TM^, and Minoza^TM^ on anti-HSV.

Extract	HSV-1 KOS		HSV-1 dxpIII		HSV-2	
	${\mathrm{IC}}_{50}$ (μg/mL)	SI (Unitless)	${\mathrm{IC}}_{50}$ (μg/mL)	SI (Unitless)	${\mathrm{IC}}_{50}$ (μg/mL)	SI (Unitless)
ACV	0.283 ± 0.000	26,554	>2000	<3.757	0.360 ± 0.000	20,875
Kerra^TM^	66.543 ± 4.759	42.769	41.394 ± 0.471	68.753	72.519 ± 2.480	39.244
KS^TM^	25.476 ± 3.430	92.204	38.257 ± 0.04	61.400	67.065 ± 3.525	35.025
Minoza^TM^	187.807 ± 39.869	62.138	73.086 ± 6.682	159.669	>200	<58.35

SI stands for selective index, calculated as ${\mathrm{CC}}_{50}$ /${\mathrm{IC}}_{50}$.

**Table 3:** List of bioactive compounds in Kerra^TM^, KS^TM^, and Minoza^TM^.

Extract	Phytochemicals					
	Name/Formula			Method		Ref.
Kerra^TM^	2-Methoxy-9H-xanthen-9-one, Isorhapontigenin, Betaine, C_20_H_28_O_4_, trans-Anethole, Eicosatetraynoic acid, NP-020078, NP-003294, C_20_H_30_O_5_, and N1-(3-chlorophenyl)-2-[2-(trifluoromethyl)-4			LC-MS/MS		[29]
KS^TM^	Piperine, pellitorine, 6-gingerol, piperlonguminine, plumbagin, and 6-shogaol			UPLC-TQD/MS		[30]
Minoza^TM^	Name	Formula	*m*/*z*	RT (min)	Molecular Weight (Da)	Area
	Sargassopenilline D	$C_{19}H_{22}O_{9}$	395.133	3.56	394.12576	1.12 × 10^10^
	Parmoether A	$C_{28}H_{28}O_{11}$	541.16974	5.053	540.16249	7.94 × 10^9^
	4R-aminopentanoic acid	$C_{5}H_{11}{\mathrm{NO}}_{2}$	118.08586	0.379	117.07859	4.57 × 10^9^
	(R)-2,4,5-Trimethoxydalbergiquinol	$C_{18}H_{20}O_{3}$	285.14847	6.366	284.14119	1.11 × 10^9^
	Pseudosindorin	$C_{15}H_{12}O_{5}$	273.07559	4.477	272.06832	8.57 × 10^8^
	5,3′-Dihydroxy-6,7,4′,5′-tetramethoxyflavanone	$C_{19}H_{20}O_{8}$	377.12302	4.436	376.11574	8.30 × 10^8^
	iso-Debromo-laurinterol	$C_{15}H_{20}O$	217.15854	6.349	216.15128	8.22 × 10^8^
	2-(5-((2Z,5Z,8Z,11Z)-tetradeca-2,5,8,11-tetraen-1-yl)furan-2-yl)-ethanoic acid	$C_{20}H_{26}O_{3}$	315.19531	6.091	314.18804	6.55 × 10^8^
	Hypnosin	$C_{7}H_{7}N_{3}O_{3}$	182.05577	0.357	181.04849	5.60 × 10^8^
	4-Hydroxy-2′,4′-dimethoxydihydrochalcone	$C_{17}H_{18}O_{4}$	287.12781	6.858	286.12055	5.06 × 10^8^

UPLC-TQD/MS stands for Ultra-Performance Liquid Chromatography–Triple Quadrupole Mass Spectrometry.

**Table 4:** Binding affinity and drug-likeness prediction of candidate inhibitors; analysis by SwissADME.

Phytochemicals	Binding Energy (kcal/mol)	MW g/mol (<500)	LogP (<5)	HBA (<10)	HBD (<5)	RB (<10)	TPA Å^2^ <140	Water Solubility	Drug-Likeness
2-Methoxy-9H-xanthen-9-one	−7.0	226.23	2.85	3	0	1	39.44	Moderately soluble	Yes
Isorhapontigenin	−6.7	258.27	2.63	4	3	3	69.92	Soluble	Yes [31]
Piperine	−7.0	285.34	3.03	3	0	4	38.77	Soluble	Yes
Pellitorine	−5.4	223.35	3.64	1	1	9	29.10	Soluble	Yes
Sargassopenilline D	−6.9	394.37	0.95	9	1	4	125.43	Soluble	Yes
Parmoether A	−8.0	540.52	3.83	11	5	9	140.34	Poorly soluble	No

The symbols presented are MW, molecular weight; HBA, H-bond acceptors; HBD, H-bond donors; RB, rotatable bonds; TPA, topological polar surface area.

## Data Availability

The data that support the findings of this study are available from the corresponding author, J.C., upon reasonable request.

## Supplementary Materials

The following supporting information can be downloaded at https://www.mdpi.com/article/10.3390/v17070889/s1: Figure S1: Plaque formation of Kerra^TM^-, KS^TM^-, and Minoza^TM^-treated Vero cells infected with HSV-1 KOS and HSV-2 in post-entry step; Figure S2: Plaque formation of Kerra^TM^-, KS^TM^-, and Minoza^TM^-treated HSV-1 dxpIII-infected Vero cells in post-entry step; Figure S3: Plaque formation of Kerra^TM^, KS^TM^, and Minoza^TM^ at high MOI of HSV-1 dxpIII infection in post-entry step; Figure S4: Plaque formation of Kerra^TM^, KS^TM^, and Minoza^TM^ in HSV infection in pre-entry step; Figure S5: Effects of Kerra^TM^, KS^TM^, and Minoza^TM^ on anti-HSV-1 dxpIII in receptor-binding, viral penetration, viral attachment, and viral release assays; Figure S6: Effects of KS^TM^ on *IFNa1* and *IL1B* expressions in HSV-1 dxpIII-infected HeLa; Figure S7: Effects of 400 and 800 μg/mL Minoza^TM^ on anti-HSV-1 dxpIII in post-entry step; Table S1: Primer sequence; Table S2: Phytochemicals found in Minoza^TM^.
