# Supplementary material for: Anti-Herpes Simplex Virus (Wild-Type and Drug-Resistant) Properties of Herbal KerraTM, KSTM, and MinozaTM
Source: Viruses. 2025 Jun 24;17(7):889. doi: 10.3390/v17070889 (PMC12299012; doi:10.3390/v17070889)
Supplement: Supplementary file 1 [file viruses-17-00889-s001.zip › viruses-3704943-supplementary.pdf]

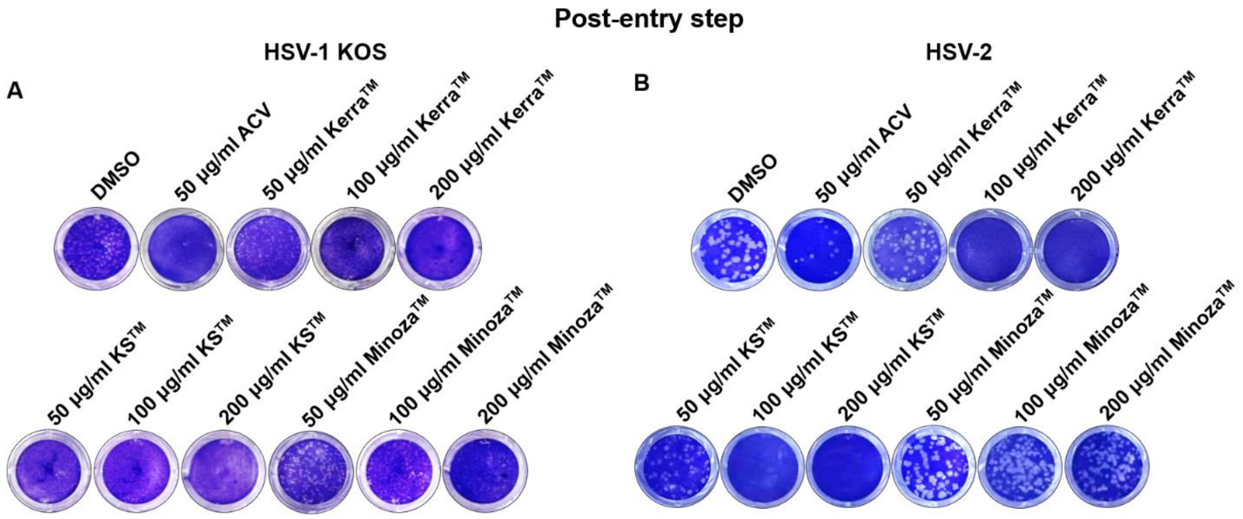

Figure S1. Plaque formation of Kerra™, KS™, and Minoza™-treated Vero cells infected with HSV-1 KOS and HSV-2 in post-entry step. Each (A) HSV-1 or (B) HSV-2 at MOI 0.002 was infected in Vero cells for 2 hours before adding the extract. Plaque formation was determined after Kerra™, KS™, and Minoza™ treatment for 48-72 hours.

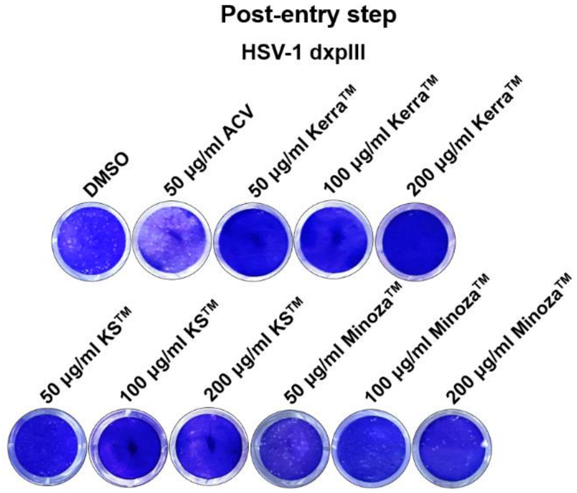

Figure S2. Plaque formation of Kerra™, KS™, and Minoza™-treated HSV-1 dxpIII-infected Vero cells in post-entry step. HSV-1 dxpIII at MOI 0.002 was infected in Vero cells for 2 hours before treatment with extracts. Plaque formation was determined after Kerra™, KS™, and Minoza™ treatment for 72 hours.

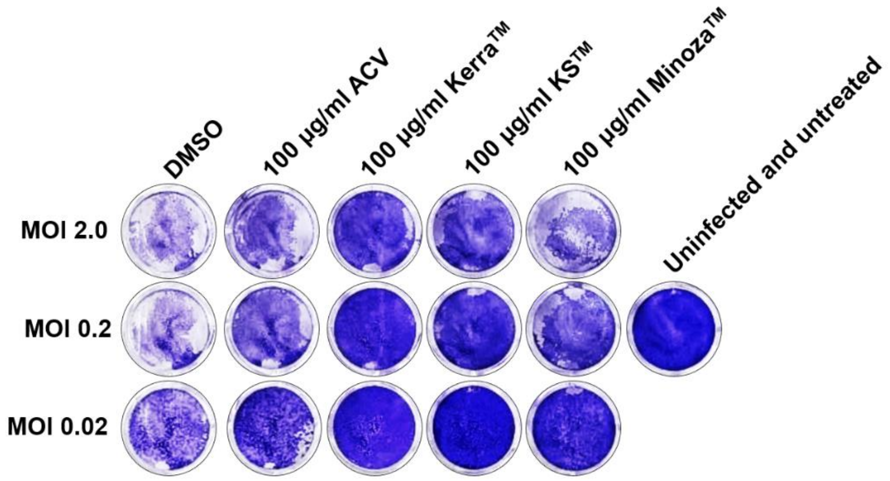

Figure S3. Plaque formation of Kerra™, KS™, and Minoza™ at high MOI of HSV-1 dxpIII infection in post-entry step. HSV-1 dxpIII at MOI 0.02, 0.2 and 2.0 were infected in Vero cells for 2 hours before treatment with the extracts. Plaque formation was determined after Kerra™, KS™, and Minoza™ treatment for 72 hours.

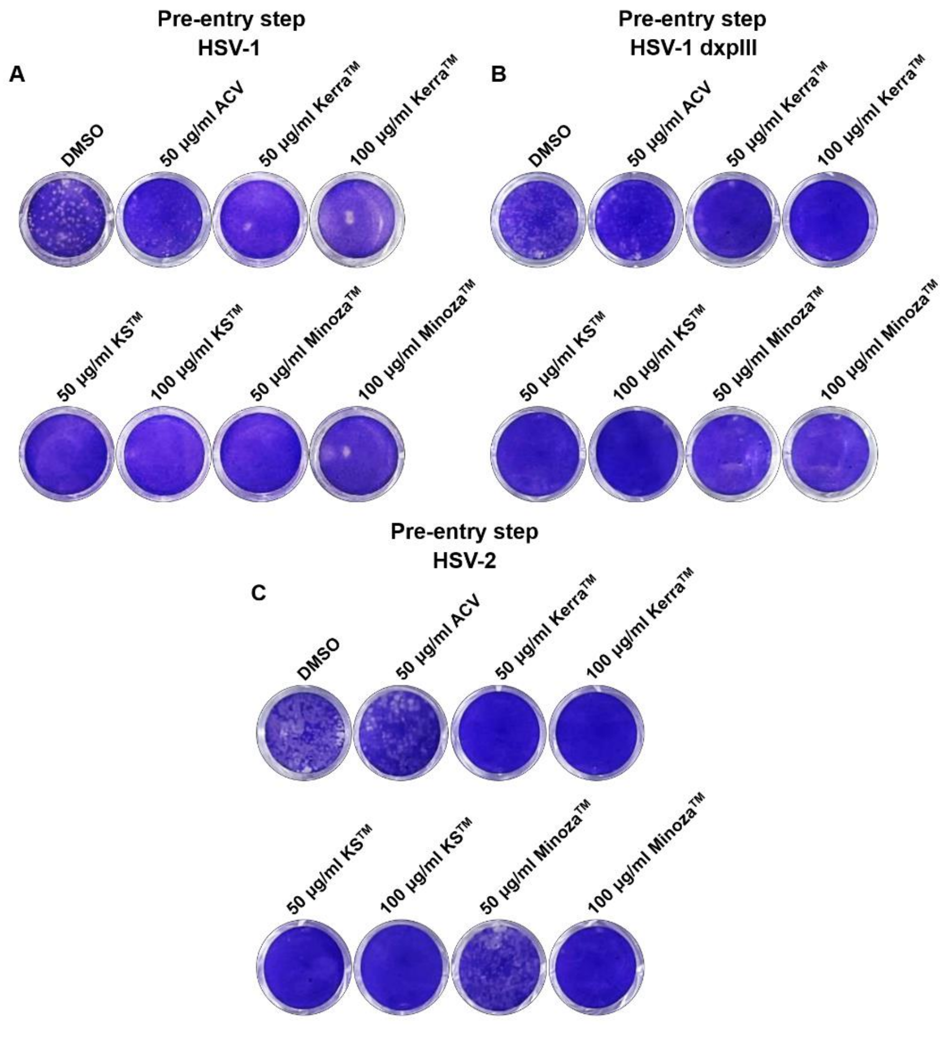

Figure S4. Plaque formation of Kerra™, KS™, and Minoza™ in HSV infection in pre-entry step. Kerra™, KS™, and Minoza™ were pre-incubated with HSV at MOI 0.002 for 1 hour before infection in Vero cells. After 48-72 hours post-infection, plaque formation was stained with crystal violet.

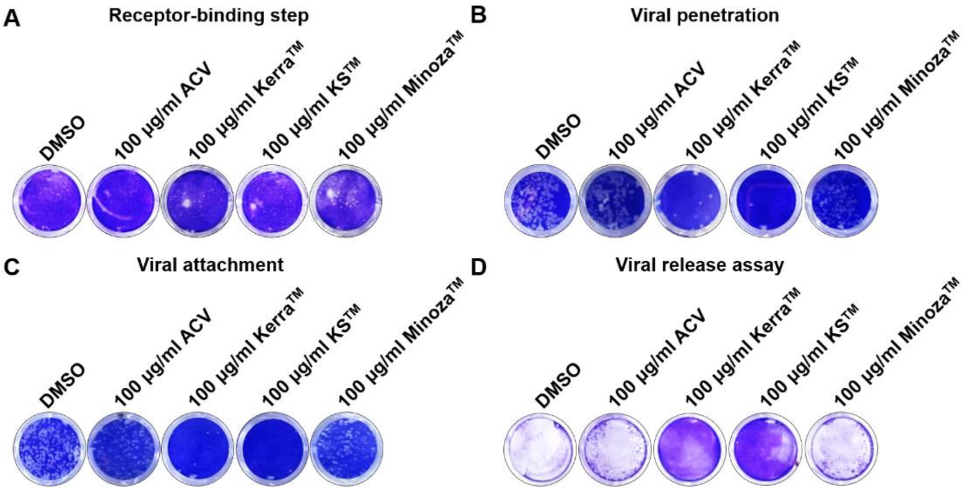

Figure S5. Effects of Kerra™, KS™, and Minoza™ on anti-HSV-1 dxpIII in receptor-binding, viral penetration, viral attachment, and viral release assays. Kerra™, KS™, and Minoza™ were subjected to Vero cells to investigate their effects on anti-HSV-1 dxpIII infection in (A) receptor-binding, (B) viral penetration, (C) viral attachment, and (D) viral release assays. After 72 hours post-infection, plaque formation was stained with crystal violet.

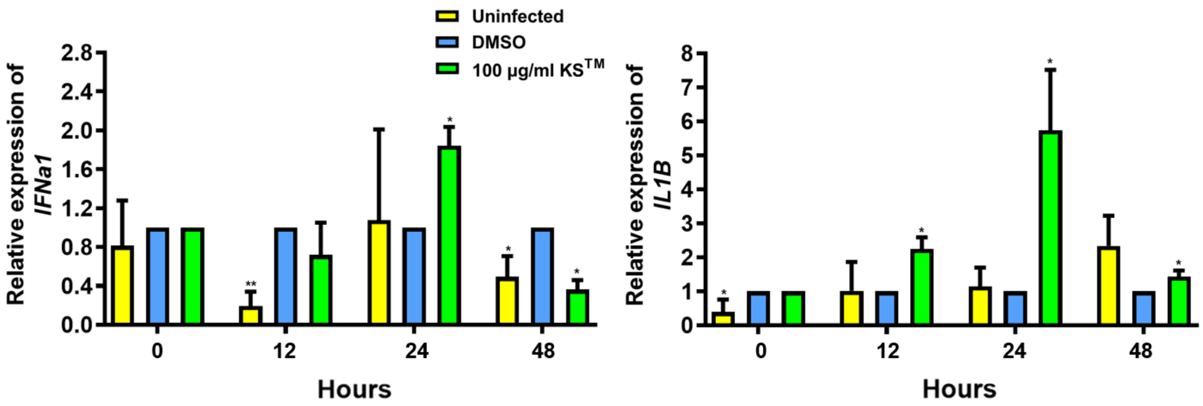

Figure S6. Effects of KS™ on *IFNa1* and *IL1B* expressions in HSV-1 dxpIII-infected HeLa. The mRNA expression levels of (**A**) *IFNa1,* and (**B**) *IL1B* were investigated in 100 μg/mL KS™-treated HeLa cells for 0–48 h. DMSO acted as a negative control. The symbols *, and ** indicate significant differences ($p < 0.05$, and 0.01, respectively). Bar charts represent the mean and SEM of triplicate experiments.

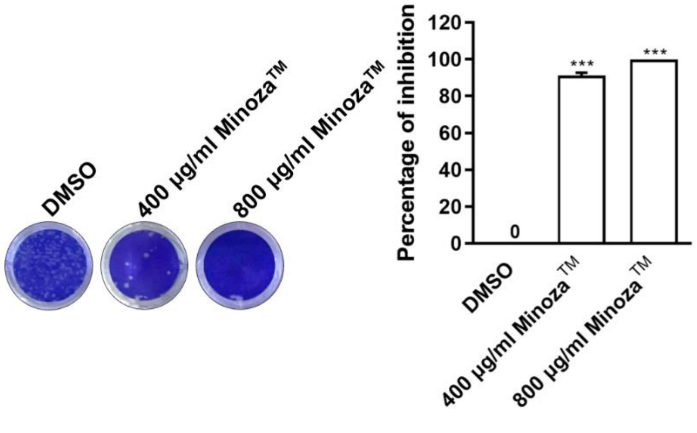

Figure S7. Effects of 400 and 800 μg/mL Minoza™ on anti-HSV-1 dxpIII in post-entry step.

Table S1. Primer sequence

Primer	Sequence	PCR product
GAPDH	Forward: 5′-TCATCAGCAATGCCTCCTGCA-3′<br>Reverse: 5′-TGGGTAGCAGTGATGGCA-3′	118 bp
gD	Forward: 5′-AGCAGGGGTTAGGGAGTTGT-3′<br>Reverse: 5′-CCATCTTGAGAGAGGCATCC-3′	212 bp
ICP4	Forward: 5′-CGACACGGATCCACGACCC-3′<br>Reverse: 5′-GATCCCCCTCCCGCGCTTCGTCCG-3′	101 bp
IL1B	Forward: 5′-GATGGCCCTAAACAGATGAAG-3′<br>Reverse: 5′-GCCTGAAGCCCTTGCT-3′	109 bp
IL6	Forward: 5′-ATGTAGCCGCCCCACACAGA-3′<br>Reverse: 5′-CATCCATCTTTTTCAGCCAT-3′	190 bp
NFKB1	Forward: 5′-GAAATTCCTGATCCAGACAAAAAC-3′<br>Reverse: 5′-ATCACTTCAATGGCCTCTGTGTAG-3′	194 bp
STAT3	Forward: 5′-CTGGCCTTTGGTGTTGAAAT-3′<br>Reverse: 5′-AAGGCACCCACAGAAACAAC-3′	202 bp
UL30	Forward: 5′-GTGTTGTGCCGCGGTCTCAC-3′<br>Reverse: 5′-GGTGAACGTCTTTTCGAACTC-3′	200 bp

Table S2. Phytochemicals found in Minoza™

Name	Formula	Annot. DeltaMass [ppm]	Calc. MW	m/z	RT [min]	Reference Ion	Area: minosa.raw (F104)	Relative amount (%)
Sargassopenilline D	C19 H22 O9	-1.58	394.1258	395.133	3.56	[M+H]+1	1.12E+10	21.0374
Parmoether A	C28 H28 O11	-1.25	540.1625	541.1697	5.053	[M+H]+1	7.94E+09	14.8784
4R-aminopentanoic acid	C5 H11 N O2	-3.35	117.0786	118.0859	0.379	[M+H]+1	4.57E+09	8.5668
(R) -2,4,5-Trimethoxydalbergiquinol	C18 H20 O3	-0.19	284.1412	285.1485	6.366	[M+H]+1	1.11E+09	2.0887
Pseudosindorin	C15 H12 O5	-0.58	272.0683	273.0756	4.477	[M+H]+1	8.57E+08	1.6055
5,3'-Dihydroxy-6,7,4',5'-tetramethoxyflavanone	C19 H20 O8	-0.21	376.1157	377.123	4.436	[M+H]+1	8.30E+08	1.5543
iso-Debromo-laurinterol	C15 H20 O	-0.62	216.1513	217.1585	6.349	[M+H]+1	8.22E+08	1.5391
2-(5-((2Z,5Z,8Z,11Z)-tetradeca-2,5,8,11-tetraen-1-yl)furan-2-yl)-ethanoic acid	C20 H26 O3	-0.5	314.188	315.1953	6.091	[M+H]+1	6.55E+08	1.2273
Hypnosin	C7 H7 N3 O3	-1.36	181.0485	182.0558	0.357	[M+H]+1	5.60E+08	1.0486
4-Hydroxy-2',4'-dimethoxydihydrochalcone	C17 H18 O4	0.16	286.1206	287.1278	6.858	[M+H]+1	5.06E+08	0.948
Hispidol 6-glucoside	C21 H20 O9	-0.58	416.1105	417.1178	5.037	[M+H]+1	4.35E+08	0.8152
6-methyl-[1,1-biphenyl]-3,3,4,5-tetraol	C13 H12 O4	-0.37	232.0735	233.0808	5.165	[M+H]+1	4.34E+08	0.8131
Rel-(2R,3R,4R)-2,3,4-trihydroxypentyl 2,4-dihydroxy-6-methylbenzoate	C13 H18 O7	-0.29	286.1052	287.1125	1.235	[M+H]+1	4.29E+08	0.8032
Pentadecanoyl-EA	C17 H35 N O2	-0.14	285.2667	286.274	8.084	[M+H]+1	3.86E+08	0.7226
5,6,15-trihydroxy-7,9,13-Eicosatrien-11-ynoic acid	C20 H30 O5	-0.41	350.2092	373.1984	6.093	[M+Na]+1	3.76E+08	0.7039
5,7,2'-Trihydroxyflavone 7-glucuronide	C21 H18 O11	-0.4	446.0847	447.092	5.619	[M+H]+1	3.65E+08	0.6845
Preussochromone E	C15 H16 O7	0.03	308.0896	617.1865	5.907	[2M+H]+1	3.35E+08	0.6285
Monospermoside	C21 H22 O10	-0.39	434.1211	457.1104	5.021	[M+Na]+1	3.35E+08	0.628
9-(3,5-dimethylfuran-2-yl)-nonanoic acid	C15 H24 O3	-0.57	252.1724	275.1616	6.88	[M+Na]+1	3.34E+08	0.6251

Globosuxanthone C	C14 H10 O5	-0.22	258.0528	291.0863	4.336	[M+H+MeOH]+1	3.28E+08	0.6142
4'-O-Methyldavidigenin	C16 H16 O4	-0.45	272.1047	273.112	6.335	[M+H]+1	3.25E+08	0.6098
Ceriporic acid B	C21 H38 O4	-0.39	354.2769	377.2661	10.384	[M+Na]+1	3.24E+08	0.6078
Ambanol	C19 H16 O6	-0.38	340.0946	341.1018	5.713	[M+H]+1	3.11E+08	0.5829
3'-C-Glucosylisoliquiritigenin	C21 H22 O9	-0.27	418.1263	419.1335	5.392	[M+H]+1	3.10E+08	0.5811
Japanic acid	C21 H40 O4	-0.79	356.2924	379.2816	10.691	[M+Na]+1	3.08E+08	0.5769
Desmosdumotin C	C19 H20 O4	0.19	312.1362	313.1435	7.379	[M+H]+1	2.96E+08	0.5538
2'-Hydroxy-4'-methoxy-3-phenylpropiophenone	C16 H16 O3	-0.61	256.1098	257.1171	6.751	[M+H]+1	2.87E+08	0.5379
Creolophin D	C14 H16 O4	-0.55	248.1047	249.112	4.783	[M+H]+1	2.69E+08	0.504
Coibacin A	C19 H24 O2	-0.43	284.1775	285.1848	6.091	[M+H]+1	2.69E+08	0.5039
6-hydroxytrideca-7,8-dien-10,12-diynoic acid	C13 H14 O3	-0.88	218.0941	219.1014	4.99	[M+H]+1	2.55E+08	0.4775
beta-vinyl acrylic acid	C5 H6 O2	-3.92	98.0364	116.0702	0.385	[M+NH4]+1	2.52E+08	0.4719
steviol-19-O-beta-D-glucoside	C26 H40 O8	0.05	480.2723	503.2615	7.283	[M+Na]+1	2.51E+08	0.4695
Isariketide	C14 H16 O8	-0.38	312.0844	313.0917	5.379	[M+H]+1	2.37E+08	0.4447
Nigerapyrone C	C13 H14 O4	-0.47	234.0891	235.0964	5.53	[M+H]+1	2.35E+08	0.4403
9-hydroxy-2Z,5E,7Z,11Z,14Z-Eicosapentaenoic acid	C20 H30 O3	0.01	318.2195	319.2268	7.285	[M+H]+1	2.28E+08	0.4269
2',4'-Dihydroxy-3,4-dimethoxychalcone	C17 H16 O5	0.01	300.0998	301.1071	4.864	[M+H]+1	2.22E+08	0.4151
Chrysoqueen	C17 H18 O10	-0.38	382.0899	383.0971	3.217	[M+H]+1	2.21E+08	0.4149
Visnagin	C13 H10 O4	-0.66	230.0578	231.065	4.335	[M+H]+1	2.19E+08	0.411
5,8,12-Trihydroxy-9-octadecenoic acid	C18 H34 O5	-0.21	330.2406	353.2298	7.692	[M+Na]+1	2.14E+08	0.4017
11-oxo-undeca-5,8-dienoic acid	C11 H16 O3	0.06	196.11	197.1173	4.767	[M+H]+1	2.09E+08	0.3925
KH01-A	C13 H22 O3	-0.93	226.1567	209.1534	6.062	[M+H-H2O]+1	2.09E+08	0.3907

Malyngic acid	C18 H32 O5	0.02	328.225	351.2142	7.381	[M+Na]+1	2.06E+08	0.3866
Psychrophilin A	C23 H20 N4 O5	-3.33	432.1419	433.1492	4.834	[M+H]+1	1.95E+08	0.3653
Isoliquiritigenin 4-methyl ether	C16 H14 O4	-0.78	270.089	271.0963	5.014	[M+H]+1	1.93E+08	0.3616
2E,6Z-farnesal	C15 H24 O	-0.53	220.1826	203.1793	8.089	[M+H-H2O]+1	1.87E+08	0.3498
2-methyl-16-heptadecenoic acid	C18 H34 O2	-0.66	282.2557	283.263	9.972	[M+H]+1	1.77E+08	0.3317
Isoliquiritigenin 4,4'-dimethyl ether	C17 H16 O4	0.04	284.1049	285.1122	5.55	[M+H]+1	1.74E+08	0.3256
3,4,4',alpha-Tetrahydroxy-2'-methoxydihydrochalcone	C16 H16 O6	-0.44	304.0946	287.0913	3.917	[M+H-H2O]+1	1.69E+08	0.3175
2-hydroxyethyl-3-methyl-1,4-naphthoquinone	C13 H12 O3	-1.02	216.0784	249.1119	5.015	[M+H+MeOH]+1	1.68E+08	0.315
Hispidol	C15 H10 O4	-0.6	254.0578	255.065	6.169	[M+H]+1	1.67E+08	0.3122
Phloretin	C15 H14 O5	-1.11	274.0838	275.0911	3.562	[M+H]+1	1.65E+08	0.3087
3,4,2',4',6'-Pentahydroxydihydrochalcone	C15 H14 O6	-0.35	290.0789	291.0862	5.044	[M+H]+1	1.64E+08	0.3078
Oleraceacid A	C15 H24 O2	-0.68	236.1775	259.1667	8.372	[M+Na]+1	1.46E+08	0.273
Oximoaspergillimide	C11 H20 N2 O3	-1.19	228.1471	261.1806	0.392	[M+H+MeOH]+1	1.44E+08	0.2694
F-11334-B1	C11 H14 O3	0.19	194.0943	195.1016	4.136	[M+H]+1	1.41E+08	0.2639
(7E,9E,15E)-heptadeca-7,9,15-trien-11,13-diyn-4-one	C17 H20 O	-0.51	240.1513	241.1586	6.365	[M+H]+1	1.40E+08	0.2618
9-phenyl nonanoic acid	C15 H22 O2	-0.68	234.1618	235.1691	6.344	[M+H]+1	1.36E+08	0.2552
1-ethyl-2-methylbenzene	C9 H12	-2.83	120.0936	121.1008	6.38	[M+H]+1	1.34E+08	0.251
15:6(2Z,4E,6Z,8E,12E,14)(6Me,8Me,10Me[S],13Me)	C19 H26 O2	0.12	286.1933	287.2006	7.199	[M+H]+1	1.34E+08	0.2503
2,6,3',4'-Tetrahydroxy-2-benzylcoumaranone	C15 H12 O6	-0.06	288.0634	289.0707	5.01	[M+H]+1	1.31E+08	0.2459
5Z,8Z,11Z,14Z-Eicosatetraenedioic acid	C20 H30 O4	0.13	334.2145	357.2037	7.201	[M+Na]+1	1.31E+08	0.2454
Diplopyrone	C10 H12 O4	-0.19	196.0735	197.0808	5.666	[M+H]+1	1.31E+08	0.2448
Benzoic acid	C7 H6 O2	-2.72	122.0365	123.0437	6.795	[M+H]+1	1.29E+08	0.242

Rhytidchromone D	C17 H20 O8	-0.31	352.1157	353.123	3.821	[M+H]+1	1.28E+08	0.2394
Gibberellin A24	C20 H26 O5	0.01	346.178	347.1853	6.657	[M+H]+1	1.27E+08	0.2385
Homobutein	C16 H14 O5	-0.01	286.0841	287.0914	5.738	[M+H]+1	1.27E+08	0.2374
Oleamide	C18 H35 N O	-0.21	281.2718	282.2791	10.033	[M+H]+1	1.17E+08	0.2192
6-Hydroxyoctadeca-7,9-diynoic acid	C18 H28 O3	0.13	292.2039	275.2006	7.378	[M+H-H2O]+1	1.16E+08	0.2179
Protofarrerol	C17 H18 O6	0.2	318.1104	301.1071	5.185	[M+H-H2O]+1	1.11E+08	0.2081
methyl 10-hydroxy-8,9-epoxy-2Z-decen-4,6-diynoate	C11 H10 O4	-0.75	206.0578	207.065	4.921	[M+H]+1	1.11E+08	0.2079
Maristachone B	C11 H16 O4	-0.35	212.1048	213.112	4.529	[M+H]+1	1.11E+08	0.2078
2S-amino-butanoic acid	C4 H9 N O2	-2.92	103.063	86.05975	0.364	[M+H-H2O]+1	1.10E+08	0.2061
3,4,2'-Trihydroxy-6",6"-dimethylpyrano [2",3":4',3'] chalcone	C20 H18 O5	-0.32	338.1153	339.1226	7.977	[M+H]+1	1.08E+08	0.2029
3,5,6-Trimethoxy-3',4'-methylene-dioxyfurano [2",3":7,8] flavanone	C21 H18 O8	-0.52	398.1	399.1072	5.037	[M+H]+1	1.08E+08	0.2021
3,5,7-Octatriyn-1-ol	C8 H6 O	-2.91	118.0415	119.0488	5.466	[M+H]+1	1.06E+08	0.1989
Sulfuretin	C15 H10 O5	-0.4	270.0527	271.06	4.482	[M+H]+1	1.05E+08	0.1976
2,4-octadienal	C8 H12 O	-2.81	124.0885	125.0957	5.592	[M+H]+1	1.04E+08	0.1953
10(R)-hydroxy-8Z-octadeceneic acid	C18 H34 O3	-0.61	298.2506	321.2398	9.894	[M+Na]+1	1.04E+08	0.1952
Elephantopinolide M	C15 H16 O5	-0.31	276.0997	277.107	5.405	[M+H]+1	1.03E+08	0.1936
Tephcalostan C	C21 H16 O7	0.13	380.0897	381.0969	5.279	[M+H]+1	9.85E+07	0.1845
p-Cymene	C10 H14	-2.55	134.1092	135.1165	5.586	[M+H]+1	9.76E+07	0.1829
4,2',6'-Trihydroxy-4'-methoxy-3',5'-dimethyldihydrochalcone	C18 H20 O5	0.26	316.1312	339.1204	7.567	[M+Na]+1	9.72E+07	0.1821
Norethindrone	C20 H26 O2	0.02	298.1933	299.2006	7.2	[M+H]+1	9.60E+07	0.1799
7-phenyl heptanoic acid	C13 H18 O2	-0.82	206.1305	207.1378	6.345	[M+H]+1	9.51E+07	0.1782
Lentialexin	C8 H8 O	-2.86	120.0572	121.0644	5.459	[M+H]+1	9.35E+07	0.1751

Acetyl-D-carnitine	C9 H17 N O4	-1.19	203.1155	236.149	0.386	[M+H+MeOH]+1	9.17E+07	0.1719
X-14847	C12 H23 N O10	-0.2	341.1321	342.1394	0.335	[M+H]+1	9.16E+07	0.1716
Suspensolide	C12 H18 O2	0.46	194.1308	195.1381	7.513	[M+H]+1	8.96E+07	0.1678
Virescenoside Z6	C26 H40 O9	0.03	496.2673	519.2565	6.514	[M+Na]+1	8.72E+07	0.1633
3-Methylsubericacid	C9 H16 O4	-0.7	188.1047	211.094	5.632	[M+Na]+1	8.71E+07	0.1632
11-Methyl-2Z,5Z-dodecadienoic acid	C13 H22 O2	-0.46	210.1619	211.1692	5.919	[M+H]+1	8.35E+07	0.1564
Bakuchalcone	C20 H20 O5	-0.23	340.131	341.1383	7.769	[M+H]+1	8.33E+07	0.156
15-deoxy-delta-12,14-PGJ2	C20 H28 O3	-0.09	316.2038	317.2111	6.512	[M+H]+1	8.28E+07	0.1551
Phytosphingosine	C18 H39 N O3	-0.11	317.293	318.3002	8.611	[M+H]+1	8.20E+07	0.1536
Okanin 3,4-dimethyl ether 4'-glucoside	C23 H26 O11	-0.2	478.1474	479.1547	4.755	[M+H]+1	8.18E+07	0.1532
FL1AAKGM0001_a	C19 H18 O7	0.14	358.1053	359.1126	4.199	[M+H]+1	7.99E+07	0.1496
Octadeca-9-en-12,14,16-triynoic acid	C18 H22 O2	-0.63	270.1618	271.169	6.092	[M+H]+1	7.83E+07	0.1467
Isoliquiritigenin	C15 H12 O4	-0.92	256.0733	257.0806	3.831	[M+H]+1	7.83E+07	0.1467
2'-Hydroxy-4',6'-dimethoxy-3'-methyldihydrochalcone	C18 H20 O4	0.1	300.1362	301.1435	5.735	[M+H]+1	7.79E+07	0.146
N-fumaryl-L-alanine dimethyl ester	C9 H13 N O5	-1.19	215.0791	248.1126	0.385	[M+H+MeOH]+1	7.76E+07	0.1453
3-pentadecynoic acid	C15 H26 O2	-0.79	238.1931	261.1823	8.368	[M+Na]+1	7.73E+07	0.1448
6,8,10,12-pentadecatetraenal	C15 H22 O	-0.34	218.167	219.1742	8.029	[M+H]+1	7.57E+07	0.1419
Heterofibrin B1	C19 H28 O2	0.14	288.209	289.2163	7.284	[M+H]+1	7.43E+07	0.1392
3-(3-methylfuran-2,5-diyl)-dipropionic acid	C11 H14 O5	-0.81	226.0839	227.0912	4.062	[M+H]+1	7.37E+07	0.1381
(3S*,4S*)-3,4-dihydroxy-7-methyl-3,4-dihydro-1(2H)-naphthalenone	C11 H12 O3	0.17	192.0787	193.086	5.263	[M+H]+1	7.28E+07	0.1363
16-methyl-6Z,9Z,12Z-heptadecatrienoic acid	C18 H30 O2	0.17	278.2246	279.2319	10.466	[M+H]+1	7.25E+07	0.1358
4,2',4'-Trihydroxy-3-methoxydihydrochalcone	C16 H16 O5	0.03	288.0998	289.1071	5.272	[M+H]+1	7.19E+07	0.1346

Penitricin D	C4 H4 O2	-3.38	84.02085	85.02812	0.382	[M+H]+1	7.18E+07	0.1345
Olivetolic acid	C12 H16 O4	-0.46	224.1048	207.1015	5.506	[M+H-H2O]+1	7.07E+07	0.1325
methyl 10-acetoxy-8,9-epoxy-2Z-decen-4,6-diynoate	C13 H12 O5	-0.51	248.0684	249.0756	4.483	[M+H]+1	7.01E+07	0.1314
8,11,14,18-Eicosatetraynoic acid	C20 H28 O2	-0.18	300.2089	301.2162	10.106	[M+H]+1	6.94E+07	0.13
FL1A1AGM0001_a	C16 H12 O4	-0.66	268.0734	269.0807	4.896	[M+H]+1	6.93E+07	0.1299
FL1C3CGS0020_a	C18 H18 O6	-0.07	330.1103	331.1176	4.441	[M+H]+1	6.93E+07	0.1298
Tricladolide A	C13 H14 O5	-0.43	250.084	251.0913	4.798	[M+H]+1	6.83E+07	0.1279
6,2'4'-Trihydroxy-2-phenylbenzofuran	C14 H10 O4	-0.69	242.0577	243.065	4.511	[M+H]+1	6.79E+07	0.1273
10E,12E-tetradecadiene-4,6-diynoic acid	C14 H16 O2	-0.15	216.115	217.1222	6.367	[M+H]+1	6.76E+07	0.1267
13-keto-9Z-octadecenoic acid	C18 H32 O3	-0.23	296.2351	319.2243	9.705	[M+Na]+1	6.73E+07	0.126
16-methyl-6Z,9Z-heptadecadienoic acid	C18 H32 O2	-0.19	280.2402	281.2475	9.678	[M+H]+1	6.72E+07	0.1259
1-(3-Methylphenyl)-ethanone	C9 H10 O	-2.39	134.0728	135.0801	6.363	[M+H]+1	6.61E+07	0.1238
2,6,3'-Trihydroxy-4'-methoxy-2-benzylcoumaranone	C16 H14 O6	-0.41	302.0789	285.0757	3.923	[M+H-H2O]+1	6.54E+07	0.1225
Butyramide	C4 H9 N O	-3.58	87.0681	88.07538	0.392	[M+H]+1	6.52E+07	0.1221
Isoneobavachalcone	C17 H14 O5	0	298.0841	299.0914	6.16	[M+H]+1	6.51E+07	0.122
2,10-dihydroxy-4,6,8-decatriynoic acid	C10 H8 O4	0.21	192.0423	193.0496	4.491	[M+H]+1	6.49E+07	0.1215
Ginkgolide A	C20 H24 O9	-0.27	408.1419	409.1492	4.073	[M+H]+1	6.48E+07	0.1214
Castillene B	C19 H18 O4	0.23	310.1206	311.1279	7.711	[M+H]+1	6.45E+07	0.1208
2-methyl-phenol	C7 H8 O	-2.79	108.0572	109.0645	6.358	[M+H]+1	6.42E+07	0.1203
Acetoacetamide	C4 H7 N O2	-2.97	101.0474	84.04413	0.39	[M+H-H2O]+1	6.15E+07	0.1153
1,3,6-Octatriene	C8 H12	-3.04	108.0936	109.1009	7.43	[M+H]+1	6.15E+07	0.1152
Gibberellin A36	C20 H26 O6	0.31	362.1731	363.1803	6.367	[M+H]+1	6.11E+07	0.1145

10E-Pentadecen-6,8-diynoic acid	C15 H20 O2	-0.84	232.1461	233.1534	6.68	[M+H]+1	6.03E+07	0.1129
5,8,11-heptadecatriynoic acid	C17 H22 O2	-0.5	258.1619	259.1691	7.201	[M+H]+1	5.92E+07	0.1109
4,8,11,14-Eicosatetraynoic acid	C20 H24 O2	0.1	296.1777	297.1849	6.649	[M+H]+1	5.91E+07	0.1108
Oxosorbicillinol	C14 H16 O5	-0.43	264.0997	247.0964	5.603	[M+H-H2O]+1	5.84E+07	0.1094
Dehydrophytosphingosine	C18 H37 N O3	-0.14	315.2773	316.2846	8.347	[M+H]+1	5.81E+07	0.1089
6-[3]-ladderane-1-hexanol	C18 H30 O	-0.71	262.2295	263.2368	10.385	[M+H]+1	5.78E+07	0.1084
Frustulosin	C12 H10 O3	0.51	202.0631	203.0704	5.564	[M+H]+1	5.78E+07	0.1083
9-hydroxy-10-Octadecen-12-ynoic acid	C18 H30 O3	0.05	294.2195	295.2268	7.694	[M+H]+1	5.61E+07	0.1051
Crotaoprostrin	C18 H18 O5	-0.16	314.1154	315.1227	5.385	[M+H]+1	5.60E+07	0.1049
2R-aminohexadecanoic acid	C16 H33 N O2	-0.61	271.251	272.2582	7.704	[M+H]+1	5.56E+07	0.1041
Gliricidol	C16 H16 O7	0.04	320.0896	321.0969	4.862	[M+H]+1	5.51E+07	0.1033
9,12-hexadecadienoic acid	C16 H28 O2	-0.75	252.2087	253.216	9.46	[M+H]+1	5.50E+07	0.1031
13-tetradecen-2,4-diyn-1-ol	C14 H20 O	-0.82	204.1513	205.1585	6.103	[M+H]+1	5.46E+07	0.1024
Torosaflavone A	C21 H20 O8	-0.12	400.1158	401.1231	6.493	[M+H]+1	5.46E+07	0.1023
JBIR-68	C19 H30 N2 O6	4.04	382.2119	383.2192	10.106	[M+H]+1	5.38E+07	0.1008
Lycocasuarinen acid C	C12 H18 O3	-0.13	210.1256	211.1328	6.374	[M+H]+1	5.37E+07	0.1005
3E,9Z,12Z,15Z-octadecatetraenoic acid	C18 H28 O2	-0.21	276.2089	277.2162	9.541	[M+H]+1	5.34E+07	0.1
Habiterpenol	C26 H38 O	1.3	366.2927	367.3	10.95	[M+H]+1	5.18E+07	0.0971
Zearalenone	C18 H22 O5	-0.12	318.1467	319.154	5.737	[M+H]+1	5.17E+07	0.0968
2,4,7-tridecatrienal	C13 H20 O	-0.24	192.1514	193.1587	5.403	[M+H]+1	5.14E+07	0.0963
CJ-16,170	C19 H21 N O3	0.21	311.1522	312.1595	6.602	[M+H]+1	4.97E+07	0.0931
Ambrettolic acid	C16 H30 O3	-0.69	270.2193	293.2086	9.249	[M+Na]+1	4.89E+07	0.0915

Berkazaphilone A	C13 H16 O3	-0.96	220.1097	221.117	8.123	[M+H]+1	4.88E+07	0.0914
Artoindonesianin P	C20 H16 O7	-0.29	368.0895	369.0968	4.69	[M+H]+1	4.87E+07	0.0913
5-hydroxymethylfurfural	C6 H6 O3	-2.6	126.0314	127.0386	0.672	[M+H]+1	4.85E+07	0.0909
1-methyl-pseudouridine	C10 H14 N2 O6	-1.16	258.0849	259.0922	0.39	[M+H]+1	4.73E+07	0.0887
2'-amino-2'-deoxyadenosine	C10 H14 N6 O3	0.27	266.1128	267.1201	5.881	[M+H]+1	4.72E+07	0.0884
6-amino-hexanoic acid	C6 H13 N O2	-3.41	131.0942	132.1015	0.404	[M+H]+1	4.63E+07	0.0868
DGMG(16:0/0:0)	C31 H58 O14	0.09	654.3827	677.372	10.062	[M+Na]+1	4.62E+07	0.0866
9Z,11E,13-Tetradecatrienal	C14 H22 O	-1.04	206.1669	207.1741	8.927	[M+H]+1	4.59E+07	0.086
Dichloroacetic acid	C2 H2 Cl2 O2	-0.65	127.9431	128.9504	0.585	[M+H]+1	4.54E+07	0.085
Anserinone B	C11 H14 O4	-1.04	210.089	211.0963	4.543	[M+H]+1	4.46E+07	0.0836
4-Prenyldihydropinosylvin	C19 H22 O2	-0.02	282.162	315.1955	6.647	[M+H+MeOH]+1	4.45E+07	0.0834
Tridecanamide	C13 H27 N O	-0.62	213.2091	214.2164	9.603	[M+H]+1	4.39E+07	0.0823
Corticrocin	C14 H14 O4	-0.7	246.089	247.0963	5.747	[M+H]+1	4.38E+07	0.0821
2,5-didehydro-D-gluconic acid	C6 H8 O7	-0.94	192.0268	215.0161	0.553	[M+Na]+1	4.36E+07	0.0817
Aspercyclide B	C24 H28 O6	-0.08	412.1886	413.1958	7.639	[M+H]+1	4.23E+07	0.0793
Fumiquinazoline A	C24 H23 N5 O4	-3.29	445.1735	446.1808	5.894	[M+H]+1	4.22E+07	0.079
Tetradecan-7,9-diynoic acid	C14 H20 O2	-0.68	220.1462	221.1535	6.952	[M+H]+1	4.17E+07	0.0781
2E,4E,6Z,8Z-Decatetraenedioic acid	C10 H10 O4	-0.74	194.0578	195.0652	4.605	[M+H]+1	4.10E+07	0.0769
Neobanol	C18 H12 O6	0.28	324.0635	325.0707	5.28	[M+H]+1	4.08E+07	0.0765
13Z,17-Octadecadiene-9,11-diynoic acid	C18 H24 O2	-0.23	272.1776	273.1848	6.867	[M+H]+1	4.06E+07	0.0761
Cephalosporolide J	C10 H16 O5	-0.68	216.0996	217.1069	4.179	[M+H]+1	4.04E+07	0.0757
4E,6E,10Z-Hexadecatrien-1-ol	C16 H28 O	-0.43	236.2139	237.2212	10.284	[M+H]+1	3.93E+07	0.0736

FL1AUNGS0001_a	C15 H14 O4	-0.43	258.0891	259.0964	5.497	[M+H]+1	3.88E+07	0.0726
Myristamide	C14 H29 N O	-0.82	227.2247	228.232	9.67	[M+H]+1	3.87E+07	0.0725
alpha-curcumene	C15 H22	-1.05	202.1719	203.1792	9.434	[M+H]+1	3.86E+07	0.0723
2,4-heptadienal	C7 H10 O	-3.72	110.0728	111.08	3.723	[M+H]+1	3.86E+07	0.0722
Corynechromone I	C14 H14 O6	-0.26	278.079	279.0862	5.107	[M+H]+1	3.85E+07	0.0721
2',4'-Dihydroxydihydrochalcone	C15 H14 O3	-0.79	242.0941	243.1014	6.593	[M+H]+1	3.81E+07	0.0715
Geoxantether A	C17 H18 O2	-0.57	254.1305	255.1378	6.086	[M+H]+1	3.81E+07	0.0714
Ceriporic acid C	C21 H36 O4	-0.06	352.2613	375.2505	10.104	[M+Na]+1	3.75E+07	0.0703
Prolipyrone C	C12 H13 N O5	-0.92	251.0791	252.0864	3.662	[M+H]+1	3.74E+07	0.0701
Pestalotine A	C14 H14 O7	-0.49	294.0738	295.0811	5.394	[M+H]+1	3.72E+07	0.0696
Dodecanamide	C12 H25 N O	-0.2	199.1936	200.2009	9.874	[M+H]+1	3.72E+07	0.0696
1-Phenylheptane-1,5-dione	C13 H16 O2	-0.48	204.1149	205.1222	7.286	[M+H]+1	3.71E+07	0.0694
Penidilamine	C10 H11 N O4	-1.48	209.0685	210.0758	0.393	[M+H]+1	3.67E+07	0.0687
Cacalol	C15 H16 O2	-1.1	228.1148	229.1221	6.085	[M+H]+1	3.66E+07	0.0685
2E,4E,8Z,10E-dodecatetraenoic acid	C12 H16 O2	-0.12	192.115	193.1223	7.105	[M+H]+1	3.66E+07	0.0685
9,12,15-Octadecatrien-1-ol	C18 H32 O	-1	264.2451	265.2523	10.691	[M+H]+1	3.62E+07	0.0678
Prolipyrone B	C10 H10 O5	-1.14	210.0526	211.0599	4.739	[M+H]+1	3.62E+07	0.0677
Minabeolide-4	C27 H38 O3	-0.31	410.282	411.2893	9.475	[M+H]+1	3.58E+07	0.0671
3,7-Dimethyl-2E,6E-decadien-1,10-dioic acid	C12 H18 O4	-0.97	226.1203	209.117	5.868	[M+H-H2O]+1	3.58E+07	0.0671
Leptosidin	C16 H12 O6	-0.36	300.0633	301.0706	5.396	[M+H]+1	3.57E+07	0.0668
Methylmalonylcarnitine	C11 H19 N O6	-0.73	261.1211	294.1546	0.392	[M+H+MeOH]+1	3.56E+07	0.0666
2',4'-Dihydroxy-3,4,6'-trimethoxydihydrochalcone	C18 H20 O6	0.11	332.126	333.1333	6.492	[M+H]+1	3.54E+07	0.0662

3',4'-Methylenedioxyfurano [2'',3'':6,7] aurone	C18 H10 O5	-0.36	306.0527	307.0599	5.038	[M+H]+1	3.49E+07	0.0654
all-trans-7-hydroxyhexadeca-2,4,8,10-tetraenoic acid	C16 H24 O3	-0.72	264.1724	265.1797	6.001	[M+H]+1	3.43E+07	0.0643
N-isobutylacetamide	C6 H13 N O	-3.74	115.0993	116.1066	2.895	[M+H]+1	3.39E+07	0.0635
Acremine S	C12 H14 O3	-0.41	206.0942	207.1015	5.948	[M+H]+1	3.36E+07	0.063
Hamilcone	C18 H18 O7	-0.12	346.1052	347.1125	5.391	[M+H]+1	3.36E+07	0.0629
(2'S)-2-(propan-2'-ol)-5-hydroxy-benzopyran-4-one	C12 H12 O4	-0.81	220.0734	221.0807	5.872	[M+H]+1	3.35E+07	0.0628
Valanimycin	C7 H12 N2 O3	2.32	172.0852	173.0925	6.363	[M+H]+1	3.34E+07	0.0625
3,7-Dimethyl-8,11-dioxo-2E,6E,9E-dodecatrienal	C14 H18 O3	-0.38	234.1255	235.1328	6.368	[M+H]+1	3.25E+07	0.0608
Ipomeamaronol	C15 H22 O4	-0.59	266.1517	267.1589	5.905	[M+H]+1	3.23E+07	0.0605
(6S)-dehydrovomifoliol	C13 H18 O3	-1.09	222.1254	223.1326	4.617	[M+H]+1	3.22E+07	0.0604
2-Methoxyestradiol-17beta	C19 H26 O3	-0.32	302.1881	303.1954	6.41	[M+H]+1	3.22E+07	0.0604
FL1AAAGM0001_a	C16 H12 O5	-0.13	284.0684	285.0757	5.726	[M+H]+1	3.21E+07	0.0601
7-hydroxy-10E,16-heptadecadien-8-ynoic acid	C17 H26 O3	-0.14	278.1882	261.1848	6.946	[M+H-H2O]+1	3.19E+07	0.0598
Carbazomycin D	C17 H19 N O3	0.21	285.1366	286.1438	8.039	[M+H]+1	3.18E+07	0.0596
Nocazoline A	C10 H11 N O3	-0.04	193.0739	194.0812	3.663	[M+H]+1	3.13E+07	0.0586
12-Oxo-20-carboxy-leukotriene B4	C20 H28 O6	0.33	364.1887	365.196	5.752	[M+H]+1	3.11E+07	0.0583
Azicemicin B	C22 H23 N O9	-0.13	445.1372	446.1445	4.327	[M+H]+1	3.08E+07	0.0577
Aspergiloid G	C20 H24 O3	0.06	312.1726	313.1798	5.827	[M+H]+1	3.07E+07	0.0576
Tolyporphin I	C28 H26 N4 O6	-3.57	514.1834	515.1907	5.392	[M+H]+1	3.06E+07	0.0574
9,10-Dihydro-10- (4-hydroxyphenyl) -pyrano [2,3-h] epicatechin-8-one	C24 H20 O8	0.03	436.1158	437.1231	5.334	[M+H]+1	3.00E+07	0.0562
Vinyl acetic acid	C4 H6 O2	-3.33	86.03649	87.04377	0.362	[M+H]+1	2.97E+07	0.0557
5-((E)-hept-4-en-2-yne-1-one-1-yl)-2-furanacrylic acid	C14 H12 O4	-0.6	244.0734	245.0807	5.907	[M+H]+1	2.97E+07	0.0556

Xanthoangelol D	C21 H22 O5	0.49	354.1469	355.1542	7.209	[M+H]+1	2.96E+07	0.0554
Ginsenoyne C	C17 H24 O3	-0.28	276.1725	277.1798	7.888	[M+H]+1	2.95E+07	0.0554
7alpha-Acetoxydehydrobotrydienal	C17 H20 O4	0.06	288.1362	289.1436	6.662	[M+H]+1	2.95E+07	0.0553
5-methyl-2-undecenoic acid	C12 H22 O2	-0.26	198.1619	199.1692	10.573	[M+H]+1	2.94E+07	0.055
3-amino-4,5,6-trihydroxy-2-methoxy-5-methyl-2-cyclohexen-1-one	C8 H13 N O5	-1.28	203.0791	204.0864	0.396	[M+H]+1	2.83E+07	0.053
Asperitaconic acid A	C12 H20 O5	-0.56	244.1309	245.1382	5.478	[M+H]+1	2.80E+07	0.0525
1,7-Hydroxy-3-methylxanthone	C14 H10 O3	-0.36	226.0629	227.0702	5.605	[M+H]+1	2.78E+07	0.0521
5'-deoxyguanosine	C10 H13 N5 O4	1.26	267.0971	268.1037	0.387	[M+H]+1	2.75E+07	0.0516
Farfugin A	C15 H18 O	-0.88	214.1356	215.1429	7.187	[M+H]+1	2.72E+07	0.051
Bolekolic acid	C18 H26 O3	0.5	290.1883	291.1956	7.848	[M+H]+1	2.72E+07	0.051
Grandiflorone	C19 H22 O4	-0.03	314.1518	337.141	6.021	[M+Na]+1	2.70E+07	0.0506
2,10-dimethyl 4-hydroxy-6-oxo-4-undecen-7-yne	C13 H20 O2	-0.35	208.1463	209.1535	7.559	[M+H]+1	2.64E+07	0.0494
(-)-Picrotoxinin	C15 H16 O6	-0.02	292.0947	293.102	5.749	[M+H]+1	2.62E+07	0.049
5,7,9,11,13-tetradecapentaenoic acid	C14 H18 O2	-0.5	218.1306	219.1379	7.281	[M+H]+1	2.60E+07	0.0486
Carbazomycin A	C16 H17 N O2	-0.28	255.1259	256.1331	6.307	[M+H]+1	2.56E+07	0.048
2,4,6-octatrienal	C8 H10 O	-2.66	122.0728	123.0801	5.613	[M+H]+1	2.56E+07	0.048
3'-Angeloyloxy-2',4'-dihydroxy-6'-methoxychalcone	C21 H20 O6	0.01	368.126	369.1333	8.042	[M+H]+1	2.53E+07	0.0474
4E,6E,12Z-Tetradecatriene-8,10-diynoic acid	C14 H14 O2	-1.06	214.0992	215.1064	6.091	[M+H]+1	2.39E+07	0.0448
Nematophin	C16 H20 N2 O2	-1.33	272.1521	273.1594	3.493	[M+H]+1	2.39E+07	0.0447
Brosimacutin H	C20 H24 O6	0.13	360.1573	361.1646	5.954	[M+H]+1	2.37E+07	0.0443
Anandamide (20:2, n-6)	C22 H41 N O2	-0.26	351.3136	352.3209	10.228	[M+H]+1	2.34E+07	0.0438
Glutarylcarnitine	C12 H21 N O6	-0.55	275.1367	276.144	0.395	[M+H]+1	2.32E+07	0.0435

MG(16:0/0:0/0:0)[rac]	C19 H38 O4	-0.15	330.277	313.2737	10.058	[M+H-H2O]+1	2.28E+07	0.0427
FL64DBGM0001_a	C19 H22 O5	0.34	330.1468	331.1541	5.696	[M+H]+1	2.26E+07	0.0423
2,5-dimethoxystilbene	C16 H16 O2	-0.67	240.1149	241.1222	6.09	[M+H]+1	2.24E+07	0.042
2',4',6'-Trihydroxy-3'-prenyldihydrochalcone	C20 H22 O4	-0.18	326.1518	327.159	5.637	[M+H]+1	2.20E+07	0.0411
S56-P1	C16 H26 N6 O9 S	-1.76	478.1474	479.1546	4.031	[M+H]+1	2.19E+07	0.041
TAN-950 A	C6 H8 N2 O4	2.11	172.0488	173.0561	5.982	[M+H]+1	2.19E+07	0.0409
Styrolide B	C13 H10 O3	-0.95	214.0628	215.0701	4.686	[M+H]+1	2.18E+07	0.0409
Safynol	C13 H12 O2	-0.32	200.0837	201.0909	6.081	[M+H]+1	2.18E+07	0.0409
Penicillone A	C14 H18 O4	-0.88	250.1203	233.117	5.02	[M+H-H2O]+1	2.15E+07	0.0402
Prototenellin B	C18 H19 N O4	0.06	313.1314	314.1387	5.452	[M+H]+1	2.14E+07	0.0401
10-Hydroxy-3,7-dimethyl-2E,6E-decadienoic acid	C12 H20 O3	-1.04	212.141	213.1483	7.655	[M+H]+1	2.08E+07	0.0389
3R*,4S*-1-hydroxy-3-isobutyl-4-[4-(3-methyl-2-butenyloxy)phenyl]pyrrolidine-2,5-dione	C19 H25 N O4	0.1	331.1784	332.1857	7.505	[M+H]+1	2.05E+07	0.0384
9-Keto heptadecylic acid	C17 H32 O3	-0.18	284.2351	285.2424	9.248	[M+H]+1	2.04E+07	0.0383
6-Acetamidotridecane	C15 H31 N O	-0.85	241.2404	242.2477	10.155	[M+H]+1	2.04E+07	0.0383
Sevadicin	C23 H26 N4 O4	-3.29	422.194	445.1832	5.466	[M+Na]+1	2.01E+07	0.0377
Styrene	C8 H8	-3	104.0623	105.0696	8.082	[M+H]+1	1.98E+07	0.0372
Gallicynoic acid A	C14 H22 O4	-0.22	254.1518	277.141	7.221	[M+Na]+1	1.97E+07	0.0369
Colletotric acid	C28 H28 O10	-0.21	524.1681	525.1754	6.11	[M+H]+1	1.95E+07	0.0365
Trans-Non-2-en-(4.6.8)-triyn-1-ol	C9 H6 O	-2.88	130.0415	131.0488	5.468	[M+H]+1	1.94E+07	0.0364
3,5,7-nonatriyn-1-ol	C9 H8 O	-3.02	132.0571	133.0644	6.948	[M+H]+1	1.93E+07	0.0362
Incrustoporin	C13 H14 O2	-0.21	202.0993	203.1066	6.093	[M+H]+1	1.93E+07	0.0361
4,6,11-hexadecatrienal	C16 H26 O	-1.23	234.1981	235.2054	9.587	[M+H]+1	1.90E+07	0.0355

6beta-hydroxytestosterone	C19 H28 O3	-0.3	304.2038	305.211	6.183	[M+H]+1	1.89E+07	0.0354
4R,12S-dihydroxy-9-oxo-5E,7Z,10Z,14Z-prostatetraenoic acid-cyclo[8,12]	C20 H28 O5	0.21	348.1938	349.2011	6.579	[M+H]+1	1.85E+07	0.0347
Lophachinin D	C20 H26 O4	0.76	330.1834	313.1798	7.739	[M+H-H2O]+1	1.83E+07	0.0343
Lesquerolic acid	C20 H38 O3	-1.04	326.2818	327.289	10.799	[M+H]+1	1.83E+07	0.0342
Undecanedioic acid	C11 H20 O4	-0.79	216.136	217.1433	5.636	[M+H]+1	1.83E+07	0.0342
Penidienone	C14 H18 O	-0.71	202.1356	203.1429	6.956	[M+H]+1	1.79E+07	0.0334
S-aminomethyldihydrolipoamide	C9 H20 N2 O S2	3.09	236.1024	237.1097	6.745	[M+H]+1	1.78E+07	0.0334
(14R)-oxoglyantrypine	C20 H14 N4 O3	-3.85	358.1052	359.1125	5.998	[M+H]+1	1.77E+07	0.0331
4-amino-4-cyano-butanoic acid	C5 H8 N2 O2	-3.19	128.0582	129.0654	0.362	[M+H]+1	1.77E+07	0.0331
7-Isopropenyl-4-methyl-6,7-dihydro-1-azu	C15 H16 O	-0.56	212.12	213.1273	6.374	[M+H]+1	1.76E+07	0.033
Isoheptadecasphinganine	C17 H37 N O2	-0.01	287.2824	288.2897	7.636	[M+H]+1	1.74E+07	0.0327
3',5,5'-Trihydroxy-4',6,7,8-tetramethoxyflavone	C19 H18 O9	-0.16	390.095	391.1023	7.253	[M+H]+1	1.74E+07	0.0326
Palmitoleoyl-EA	C18 H35 N O2	-0.4	297.2667	280.2634	9.646	[M+H-H2O]+1	1.72E+07	0.0323
Not named	C11 H11 N O3	-0.78	205.0737	206.081	4.284	[M+H]+1	1.72E+07	0.0323
9,10-dihydroxy-2-decenoic acid	C10 H18 O4	0.03	202.1205	203.1278	5.637	[M+H]+1	1.72E+07	0.0323
Anandamide (20:1, n-9)	C22 H43 N O2	-0.34	353.3293	376.3185	10.57	[M+Na]+1	1.69E+07	0.0316
6E-Octene-2,4-diynoic acid	C8 H6 O2	-1.38	134.0366	269.0806	0.442	[2M+H]+1	1.68E+07	0.0314
4-Hydroxyfurano [2'',3'':6,7] aurone	C17 H10 O4	0.11	278.0579	279.0652	5.27	[M+H]+1	1.66E+07	0.031
Endertiin A	C30 H44 O3	0.57	452.3293	453.3366	7.065	[M+H]+1	1.64E+07	0.0306
4-Hydrocinnamoyl-2,2,5-trimethyl-4-cyclopentene-1,3-dione	C17 H18 O3	-0.22	270.1255	271.1328	5.619	[M+H]+1	1.61E+07	0.0302
7,11-Dimethyl-3-methylene-1,6E,10-dodecatriene	C15 H24	-0.87	204.1876	205.1949	9.653	[M+H]+1	1.59E+07	0.0298

Muconic dialdehyde	C6 H6 O2	-3.43	110.0364	111.0437	0.419	[M+H]+1	1.58E+07	0.0297
2',4',3,4,alpha-Pentahydroxydihydrochalcone 3'-C-xyloside	C20 H22 O10	-0.01	422.1213	423.1286	4.454	[M+H]+1	1.58E+07	0.0296
6E,8E,14E-Hexadecatriene-10,12-diynoic acid	C16 H18 O2	-0.57	242.1305	243.1378	6.661	[M+H]+1	1.56E+07	0.0293
Sophoracoumestan A	C20 H14 O5	0.18	334.0842	335.0915	5.062	[M+H]+1	1.56E+07	0.0292
MG(18:0/0:0/0:0)[rac]	C21 H42 O4	-0.68	358.3081	341.3048	10.558	[M+H-H2O]+1	1.54E+07	0.0288
Modiolide A	C10 H14 O4	0.06	198.0892	199.0965	5.861	[M+H]+1	1.54E+07	0.0288
3-hydroxyl-2',5-dimethoxy-2-methylbibenzyl	C17 H20 O3	-0.42	272.1411	273.1484	5.74	[M+H]+1	1.51E+07	0.0283
6-[1]-ladderane hexanol	C18 H28 O	-0.97	260.2138	261.221	9.801	[M+H]+1	1.48E+07	0.0277
Microsphaerophthalide B	C11 H10 O5	-3.75	222.052	445.1104	0.386	[2M+H]+1	1.46E+07	0.0273
4,8,12,15,18-eicosapentaenoic acid	C20 H30 O2	0.25	302.2247	303.2319	10.396	[M+H]+1	1.46E+07	0.0273
t17:0 phytosphingosine	C17 H37 N O3	-0.45	303.2772	304.2845	8.294	[M+H]+1	1.44E+07	0.027
N-acetyl-3,5,11,18-tetrahydroxyoctadecyl-2-amine	C20 H41 N O5	-0.15	375.2984	376.3057	9.619	[M+H]+1	1.44E+07	0.027
Aleprylic acid	C12 H20 O2	0.41	196.1464	197.1537	8.241	[M+H]+1	1.42E+07	0.0266
Cyclo(D)-Pro-(D)-Leu	C11 H18 N2 O2	-0.8	210.1367	211.1439	4.416	[M+H]+1	1.39E+07	0.0261
(S)-(+)-Abscisic acid	C15 H20 O4	-0.94	264.1359	265.1432	3.624	[M+H]+1	1.38E+07	0.0259
Euplectin	C17 H10 O5	0.07	294.0528	295.0601	5.275	[M+H]+1	1.38E+07	0.0258
Phomallenic acid A	C15 H18 O3	-0.42	246.1255	247.1328	4.868	[M+H]+1	1.36E+07	0.0255
2-Hydroxymyristoylcarnitine	C21 H41 N O5	-0.54	387.2983	388.3055	9.378	[M+H]+1	1.36E+07	0.0255
O-propanoyl-carnitine	C10 H19 N O4	-1.18	217.1312	218.1384	0.389	[M+H]+1	1.36E+07	0.0255
Banegasine	C11 H12 N2 O2	-1.5	204.0896	205.0969	0.384	[M+H]+1	1.34E+07	0.025
CJ-15,801	C9 H15 N O5	-1.07	217.0948	218.1021	0.383	[M+H]+1	1.30E+07	0.0244
Mycinonic acid III	C13 H20 O4	-0.84	240.136	241.1432	4.977	[M+H]+1	1.30E+07	0.0244

Echiguanine B	C10 H14 N6 O2	0.64	250.118	251.1253	6.331	[M+H]+1	1.30E+07	0.0244
(13E,15E,18Z,20Z)-1-Hydroxypentacosa-13,15,18,20-tetraen-11-yn-4-one 1-acetate	C27 H40 O3	-0.24	412.2977	395.2945	9.561	[M+H-H2O]+1	1.25E+07	0.0235
Actinosporin B	C25 H26 O11	-0.7	502.1472	520.181	0.412	[M+NH4]+1	1.25E+07	0.0234
11,14-eicosadienoic acid	C20 H36 O2	-0.06	308.2715	331.2606	10.692	[M+Na]+1	1.24E+07	0.0233
(4E,6E,d14:2) sphingosine	C14 H27 N O2	-1.45	241.2038	242.2111	10.2	[M+H]+1	1.24E+07	0.0233
Fusaric acid methyl ester	C11 H15 N O2	0.39	193.1104	194.1176	0.381	[M+H]+1	1.23E+07	0.023
pentanamide	C5 H11 N O	-3.35	101.0837	102.091	0.399	[M+H]+1	1.20E+07	0.0225
Cellocidin	C4 H4 N2 O2	-3.55	112.0269	113.0342	0.415	[M+H]+1	1.17E+07	0.0218
Ergosta-5,7,22E,24(28)-tetraenol	C28 H42 O	-0.55	394.3234	395.3306	10.735	[M+H]+1	1.16E+07	0.0218
18-methyl-5Z,8Z,11Z,14Z-nonadecatetraenoic acid	C20 H32 O2	0.01	304.2402	287.2369	6.748	[M+H-H2O]+1	1.16E+07	0.0217
Linoleamide	C18 H33 N O	-0.45	279.2561	280.2634	10.53	[M+H]+1	1.13E+07	0.0212
5-Hydroxy-6-methoxy-3',4'-methylenedioxyfurano [2'',3'':7,8] flavanone	C19 H14 O7	-0.15	354.0739	355.0812	6.475	[M+H]+1	1.12E+07	0.021
Phomoenamide	C14 H24 N2 O4	-0.59	284.1734	285.1807	0.39	[M+H]+1	1.09E+07	0.0204
5,14,15-trihydroxy-6,8,10,12-Eicosatetraenoic acid	C20 H32 O5	-0.04	352.225	353.2322	5.578	[M+H]+1	1.06E+07	0.0199
7-Hydroxyflavan	C15 H14 O2	-0.19	226.0993	227.1066	7.217	[M+H]+1	1.05E+07	0.0197
Trideca-1-en-3,5,7,9,11-pentayne	C13 H6	-3.28	162.0464	163.0537	0.381	[M+H]+1	1.01E+07	0.019
Xestoaminol C	C14 H31 N O	-1.17	229.2403	230.2476	9.503	[M+H]+1	9.92E+06	0.0186
O-octanoyl-R-carnitine	C15 H29 N O4	-0.37	287.2096	288.2168	5.709	[M+H]+1	9.88E+06	0.0185
(S)-5-(4-hydroxybenzoyl)-3-isobutyrylimidazolidine-2,4-dione	C14 H14 N2 O5	-0.15	290.0902	291.0975	4.542	[M+H]+1	9.87E+06	0.0185
12-oxo-5E,8E,10Z-dodecatrienoic acid	C12 H16 O3	-0.89	208.1098	209.117	6.383	[M+H]+1	9.79E+06	0.0183
FL1A98GS0001_a	C15 H10 O3	-0.34	238.0629	239.0702	7.12	[M+H]+1	9.54E+06	0.0179

5-methyl-2E-tridecenoic acid	C14 H26 O2	-0.62	226.1931	227.2004	7.869	[M+H]+1	9.35E+06	0.0175
4'-Hydroxy-2'-methoxychalcone	C16 H14 O3	-0.45	254.0942	255.1015	7.48	[M+H]+1	9.33E+06	0.0175
Epirodin A	C16 H18	-0.63	210.1407	211.148	6.365	[M+H]+1	9.29E+06	0.0174
2-oxo-5-amino-pentanoic acid	C5 H9 N O3	-3.13	131.0578	132.0651	0.358	[M+H]+1	9.12E+06	0.0171
N-?-glutamyl-boletine	C15 H26 N2 O6	4.46	330.1806	331.1878	9.071	[M+H]+1	8.93E+06	0.0167
FL1ABGGS0001_a	C16 H12 O7	-0.57	316.0581	317.0654	5.782	[M+H]+1	8.83E+06	0.0165
Palythine-serine	C11 H18 N2 O6	-0.83	274.1163	275.1235	0.396	[M+H]+1	8.76E+06	0.0164
Semiplenamide C	C20 H39 N O2	-0.37	325.298	326.3052	10.5	[M+H]+1	8.73E+06	0.0164
4,7,10-hexadecatrienoic acid	C16 H26 O2	-0.71	250.1931	251.2004	6.388	[M+H]+1	8.67E+06	0.0162
2-methyl-tridecanedioic acid	C14 H26 O4	-1.37	258.1828	259.19	10.932	[M+H]+1	8.56E+06	0.016
Panaquinquecol 6	C19 H26 O4	0.33	318.1832	319.1905	6.579	[M+H]+1	8.34E+06	0.0156
Corynechromone F	C14 H16 O6	0.09	280.0947	281.102	3.875	[M+H]+1	8.25E+06	0.0155
Tetranor-PGF1alpha	C16 H28 O5	1.14	300.194	301.2013	10.409	[M+H]+1	8.15E+06	0.0153
Glepidotin C	C19 H22 O3	0.15	298.1569	299.1642	6.372	[M+H]+1	8.15E+06	0.0153
Resveratrol	C14 H12 O3	-0.86	228.0785	229.0857	8.336	[M+H]+1	8.10E+06	0.0152
FR-900130	C4 H5 N O2	-3.45	99.03169	100.039	0.416	[M+H]+1	7.30E+06	0.0137
Deacetyldemethylanisomycin	C11 H15 N O3	-1.37	209.1049	210.1122	0.377	[M+H]+1	7.18E+06	0.0135
8S-hydroxy-2E-Decene-4,6-diynoic acid	C10 H10 O3	-0.23	178.063	196.0969	0.396	[M+NH4]+1	7.13E+06	0.0134
2',4',4''-Trihydroxy-3',6'',6''-trimethylpyrano [2'',3'':6',5'] dihydrochalcone	C21 H24 O5	0.42	356.1625	357.1698	7.204	[M+H]+1	7.08E+06	0.0133
Salbostatin	C13 H23 N O8	-1	321.1421	322.1493	0.381	[M+H]+1	7.06E+06	0.0132
4-Methoxyfurano [2'',3'':6,7] aurone	C18 H12 O4	0.33	292.0737	293.0809	5.761	[M+H]+1	7.02E+06	0.0131
3'-O-methylbatatasin III	C16 H18 O3	-0.7	258.1254	259.1327	6.359	[M+H]+1	6.97E+06	0.0131

FAHFA(3:0/2-O-22:0)	C25 H48 O4	-0.27	412.3552	413.3624	10.666	[M+H]+1	6.88E+06	0.0129
6,3',4'-Trihydroxy-4-methoxy-5-methylaurone	C17 H14 O6	0.16	314.0791	315.0864	6.334	[M+H]+1	6.82E+06	0.0128
Isocordoin	C20 H20 O3	0.52	308.1414	309.1487	7.321	[M+H]+1	6.58E+06	0.0123
Futalosine	C19 H18 N4 O7	-3.48	414.1161	415.1234	3.797	[M+H]+1	6.55E+06	0.0123
Bacillamidin G	C17 H35 N O	-1.01	269.2716	270.2789	10.548	[M+H]+1	6.53E+06	0.0122
(4E,10E,12E)-nonadec-4,10,12-trien-6,8-diyn-2-one	C19 H24 O	-0.76	268.1825	269.1898	9.481	[M+H]+1	6.52E+06	0.0122
5Z,8Z,11Z-Eicosatrienedioic acid	C20 H32 O4	-0.46	336.2299	337.2372	6.06	[M+H]+1	6.51E+06	0.0122
2',4'-Dihydroxychalcone	C15 H12 O3	-0.25	240.0786	241.0859	6.956	[M+H]+1	6.45E+06	0.0121
Pipericine	C22 H41 N O	-0.85	335.3185	336.3258	9.943	[M+H]+1	6.45E+06	0.0121
Trehalamine	C7 H12 N2 O5	-0.39	204.0745	205.0818	0.448	[M+H]+1	6.39E+06	0.012
12-amino-octadecanoic acid	C18 H37 N O2	-0.25	299.2824	300.2897	10.354	[M+H]+1	6.11E+06	0.0114
Maremycin C2	C17 H21 N3 O5 S	1.22	379.1207	380.1279	7.889	[M+H]+1	6.11E+06	0.0114
4'-O-Methylxanthohumol	C22 H24 O5	0.07	368.1624	369.1697	7.277	[M+H]+1	6.03E+06	0.0113
6-deoxyaflaquinolone D	C16 H15 N O3	-0.39	269.1051	270.1124	6.516	[M+H]+1	5.59E+06	0.0105
Cyclocarbamide B	C15 H22 N2 O4	-1.08	294.1576	295.1649	3.484	[M+H]+1	5.45E+06	0.0102
FR198248	C9 H10 O5	0.02	198.0528	199.0601	0.43	[M+H]+1	5.25E+06	0.0098
N-(7-methyloctanoyl)homoserine lactone	C13 H23 N O3	-1.16	241.1675	242.1748	4.762	[M+H]+1	5.24E+06	0.0098
Myxothiazol	C25 H33 N3 O3 S2	-1.75	487.1955	488.2028	3.851	[M+H]+1	5.08E+06	0.0095
Demethylcerdarin	C13 H10 O6	-0.67	262.0476	263.0548	0.437	[M+H]+1	5.00E+06	0.0094
3-oxo-4-pentenoic acid	C5 H6 O3	-3.21	114.0313	115.0386	0.443	[M+H]+1	4.91E+06	0.0092
N-[(Z)-hexadec-9-enoyl]glycine methyl ester (Z9-C16:1-NAGME)	C19 H35 N O3	-0.53	325.2615	326.2688	7.902	[M+H]+1	4.67E+06	0.0088

Arphamenine B	C16 H24 N4 O4	-0.15	336.1797	337.187	4.028	[M+H]+1	4.31E+06	0.0081
N-acetyl-2,4,10,17-tetrahydroxyheptadecylamine	C19 H39 N O5	-0.08	361.2828	362.2901	9.316	[M+H]+1	3.99E+06	0.0075
Isocycloheximide	C15 H23 N O4	-0.22	281.1627	282.1699	7.997	[M+H]+1	3.86E+06	0.0072
Platensimycin B3	C23 H27 N O5	0.15	397.189	398.1963	6.829	[M+H]+1	3.80E+06	0.0071
ascr#27	C22 H40 O6	-0.99	400.2821	401.2894	10.135	[M+H]+1	3.78E+06	0.0071
Siastatin B	C8 H14 N2 O5	-0.52	218.0902	219.0974	0.393	[M+H]+1	3.58E+06	0.0067
7,7-dimethyl-5Z,8Z,11Z-eicosatrienoic acid	C22 H38 O2	0.02	334.2872	335.2945	10.233	[M+H]+1	3.54E+06	0.0066
Okanin 3,4,3'-trimethyl ether 4'-glucoside	C24 H28 O11	-0.99	492.1627	493.17	0.437	[M+H]+1	2.85E+06	0.0053
podocarpic acid	C17 H22 O3	-0.06	274.1569	275.1642	5.916	[M+H]+1	2.72E+06	0.0051
6E,8E,12E,14E-Hexadecatetraen-10-ynoic acid	C16 H20 O2	-0.73	244.1462	245.1534	10.101	[M+H]+1	2.69E+06	0.005
